# Pattern and rate in the Plio-Pleistocene evolution of modern human brain size

**DOI:** 10.1038/s41598-022-15481-3

**Published:** 2022-07-02

**Authors:** Philip D. Gingerich

**Affiliations:** Museum of Paleontology, Research Museum Center, University of Michigan, 3600 Varsity Drive, Ann Arbor, MI 48108-2228 USA

**Keywords:** Biological anthropology, Palaeontology

## Abstract

Fourteen studies of brain size evolution in Plio-Pleistocene hominins published over the past fifty years show substantial long-term increase in endocranial volume (ECV) for the broad lineage leading to modern humans. The median generation-to-generation step rate for a consensus time series of ECV values, *h*_0_ = 0.15 standard deviations per generation, is almost identical to the median step rate observed in modern biological field studies. When specimens are aggregated in a series of 100 k.y. time bins to reflect the precision of their geological ages, temporal scaling identifies four successive phases of stasis and change that are significantly different from random. Phase I from about 3.2 to 2.0 million years before present is an initial phase of relative stasis. Phase II from 2.0 to 1.5 m.y. is a phase of directional brain size increase. Phase III from 1.5 to 0.7 m.y. is a second phase of stasis. Finally, Phase IV from about 0.7 m.y. to 10 k.y. is a second phase of directional increase. The tempo (rate) and the mode (stasis, random, or directional change) of an evolutionary time series are related to each other, and both are related to the time scale appropriate for analysis.

## Introduction

Large brains are a distinguishing feature of humans today, and the fossil record shows that brain size increased substantially in the 3.2-million-year lineage leading to modern humans. Many authors have studied this quantitatively^1–14^. Each of these studies included a time series of hominin endocranial volume (ECV) measurements and their corresponding geological ages. Here all fourteen studies are integrated in one consensus time series of 233 specimens to minimize uncertainty in the estimates of ECV and geological age for individual specimens.

Many rates can be calculated to represent change in an ECV time series. Each is a net rate, calculated as the difference between two natural-log ECV values divided by their separation in time. The rates are appropriately calibrated in *haldanes*—phenotypic standard deviations per generation^15,16^. Calculation of a net rate for each pair-wise combination of measured ECV values yields a full set of rates for the time series.

Two numbers summarize the information in this full set of rates. The first is the *temporal-scaling slope*, which quantifies the relationship of all rates for the series to the time scale or interval over which each rate was calculated. The second is the *temporal-scaling intercept*, which is the inferred step rate, *h*_0_, on the generation-to-generation time scale of the evolutionary process. The *haldane* (*h*_log10_) is a subscripted rate unit, expressed in standard deviations per generation, where the subscript is the log_10_ value of the number of generations in the rate denominator^17^.

The temporal-scaling slope enables an overall assessment of the mode of evolution on a spectrum ranging from (a) *stasis*, to (b) *random change*, to (c) *directional change*. The temporal-scaling intercept, *h*_0_ or the *rate of change on a one-generation time scale*, enables construction of realistically scaled models of random change necessary to identify change that is significantly different from random. Change significantly less than random, stasis, indicates a predominance of stabilizing selection in nature. Change significantly greater than random, directional change, indicates a predominance of directional selection.

There is always some uncertainty in the estimate of ECV for an individual hominin specimen, and some uncertainty in the geological age of a specimen. Both vary across specimens in the 14 studies analyzed here. To minimize these uncertainties, median values of the ECV and age for each specimen were combined and analyzed as a full consensus time series. Then, in a second analysis, ages in the time series were aggregated or binned at the scale of their inferred precision, and the binned time series was smoothed to limit the influence of outlying ECV values. Comparisons show that aggregation and smoothing affect both the tempo (rate) and the mode (stationary, random, or directional) inferred for an evolutionary sequence, which is an important take-home message from this study.

Limits on the interpretations presented here are (1) the moderate number of brain size measurements available; and (2) the accuracy of each individual brain size measurement and associated geological age. Analysis is focused on the evolution of hominin brain size, but parallel analyses of rates and scaling for skeletal measurements representing body size or brain size in relation to body size would be worth exploring. What is new here is the focus on rates of change, and on how rates and patterns of brain size evolution change with the time scale of analysis.

## Results

### Fourteen previous studies

Fourteen studies of hominin brain size evolution are analyzed individually in the Supplementary Information. The studies included ECV values for 36–200 specimens, representing in aggregate a total of 233 different fossils, and 17–88 geological ages (Supplementary Table 1). Eight of the 14 case studies started early in the late Pliocene, and covered an interval of 2.8 m.y. or more^1–8^. Six studies started later, in what is now regarded as the early Pleistocene, and covered an interval of less than 2.5 m.y.^9–14^. Studies that started earlier in geological time are longer (averaging 3.180 m.y. in duration), but do not differ significantly in long-term regression slopes or intercepts from studies that started later (which average 1.873 m.y. in duration). All 14 studies are consistent in having relatively low long-term evolutionary rates of about 0.00011 standard deviations per generation, spanning intervals averaging 127,200 generations for the longer studies and 74,900 generations for the shorter studies.

Temporal scaling slopes and intercepts for rates in the 14 time series analyzed in Supplementary Information are listed in Supplementary Table 2. Note that the 14 step rates for hominin brain size evolution in Supplementary Table 2 are much higher than the 14 long-term rates for hominin brain size evolution in Supplementary Table 1. The rates differ by more than three orders of magnitude, which is a reminder that rate denominators are important and must be considered in any comparison of rates^17^.

### Consensus time series of hominin brain-size evolution

The 233-specimens in the aggregated consensus time series analyzed here are listed in Supplementary Dataset 1. This dataset was constructed by combining endocranial volumes and ages for all specimens in the 14 studies described in the Supplementary Information, which yielded a 1711-line dataset (Supplementary Dataset 2). Specimen names and numbers were edited to make them consistent from study to study, then entries were sorted by consensus names and the original study number. Finally, the full dataset was reduced to the 233-specimen consensus dataset by calculating a median ECV value and median age for each specimen. Medians have the advantage of minimizing outliers in the assessments of different authors.

**Figure 1:**
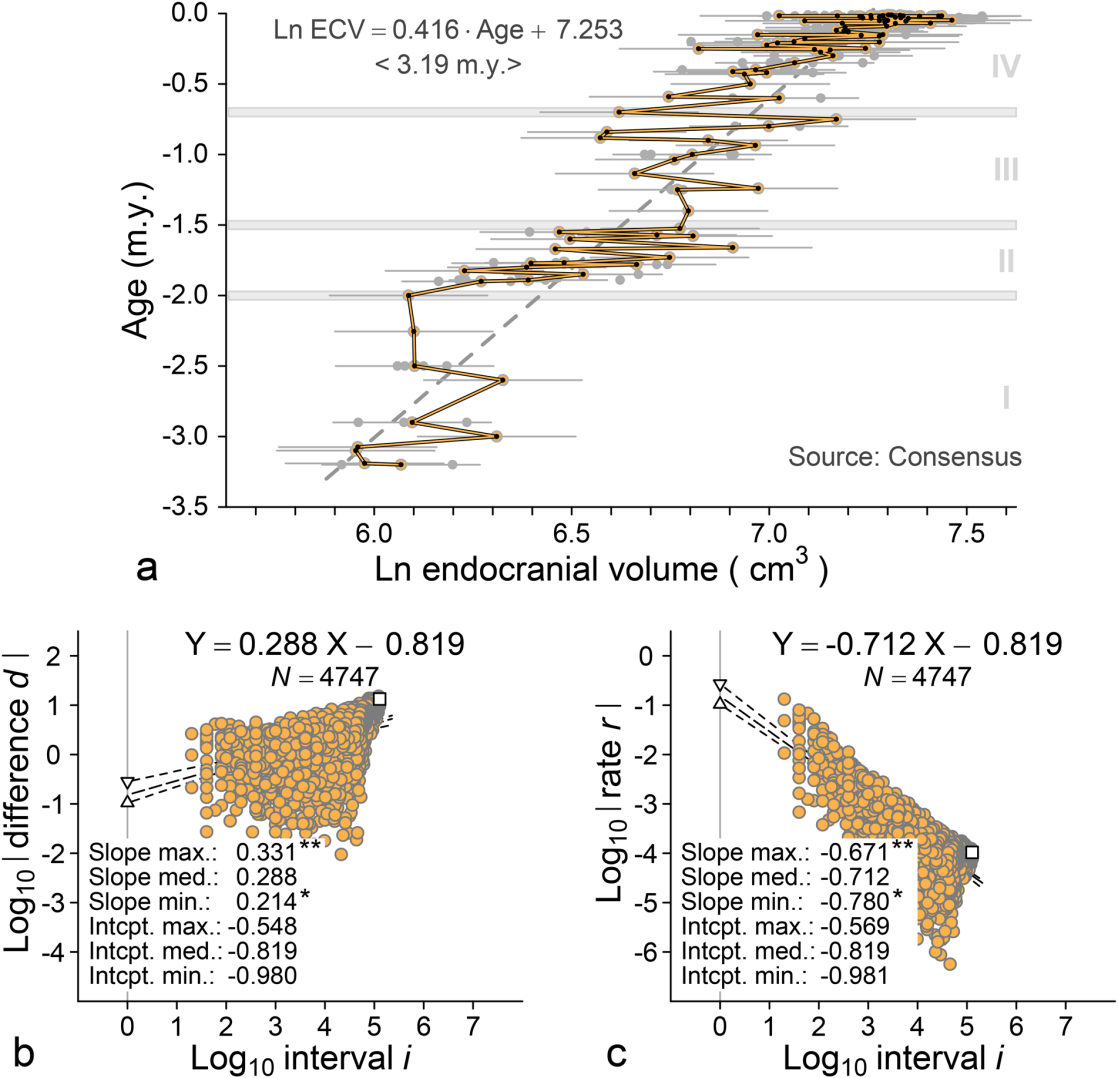
Brain size evolution in the broad lineage of Plio-Pleistocene hominins leading to modern humans. (**a**) Consensus pattern of change in endocranial volume for 233 hominin specimens representing 98 geological ages (Supplementary Dataset 1). Solid yellow line traces mean ECV values through successive ages. Regression of ln endocranial volume on geological age (dashed line) yields a single long term rate of 0.416 ln units per million years (ca. 1.041 × 10^−4^ standard deviations per generation) on a time scale of 3.19 m.y. (127,600 generations). Four phases of stasis and change, I–IV identified in Fig. 3, are shown in the background. (**b**) Log-difference-interval or LDI temporal scaling plot of change per unit time for all pairwise combinations of differences (in standard deviation units) and associated intervals (in generations) separating samples in the 98-age time series. (**c**) Log-rate-interval or LRI temporal scaling plot of change per unit time for all pairwise combinations of rates (in standard deviations per generation) and associated intervals (in generations) separating samples in the 98-age time series. Note that LDI and LRI slopes of 0.288 and − 0.712 differ by one unit: each indicates a time series more stationary than expected for purely random change. LDI and LRI intercepts are equal and indicate a step rate of 10^−0.819^ = 0.152 standard deviations per generation on a time scale of one generation. The long-term difference and the long-term rate of change represented by the dashed line in panel *a* are plotted as open squares in panels *b* and *c*.

The consensus time series is shown in Fig. 1a, where 233 specimens from 98 geological ages span an interval of 3.19 m.y. Here the independent variable, geological age (m.y.), is on the vertical axis, and the dependent variable, ln ECV (cm^3^), is on the horizontal axis. Regression of ln ECV on age for the entire time series yields a long-term slope or rate of ln ECV (cm^3^)/age (m.y.) = 0.416. Converting to standard deviation units and generations, this is a long-term rate *R* = 4.164 std. dev./40,000 gen. = 0.00011 std. dev./gen., on a time scale or interval *I* = 3,190,000/25 = 127,600 generations. The difference *D* that corresponds to *R* and *I* is *D* = *R* · *I* = 13.282 standard deviations. The intercept of ln ECV = 7.253, exponentiated, corresponds to an ECV of *h*_0_ = *e*^7.253^ = 1412 cm^3^. Regression of ln ECV on age (dashed line in Fig. 1a) is a form of aggregation and smoothing (see below).

All of the possible differences between sample mean ECVs in the time series of Fig. 1a (expressed in standard deviation units) are plotted against their corresponding intervals (expressed in generations) on the log-difference-interval or LDI graph of Fig. 1b. The 4747 log differences range in value from − 3.763 to 1.179, on log intervals ranging from 1.301 to 5.106. The distribution of differences has a median slope of 0.288. Asterisks at range limits indicate statistical significance at *p* < 0.05. The slope of 0.288 in Fig. 1b is significantly different from the slope of 1.000 expected for directional change and from 0.500 expected for random change (hence the two asterisks); 0.288 is also significantly different from the slope of 0.000 expected for stasis (single asterisk).

**Figure 2:**
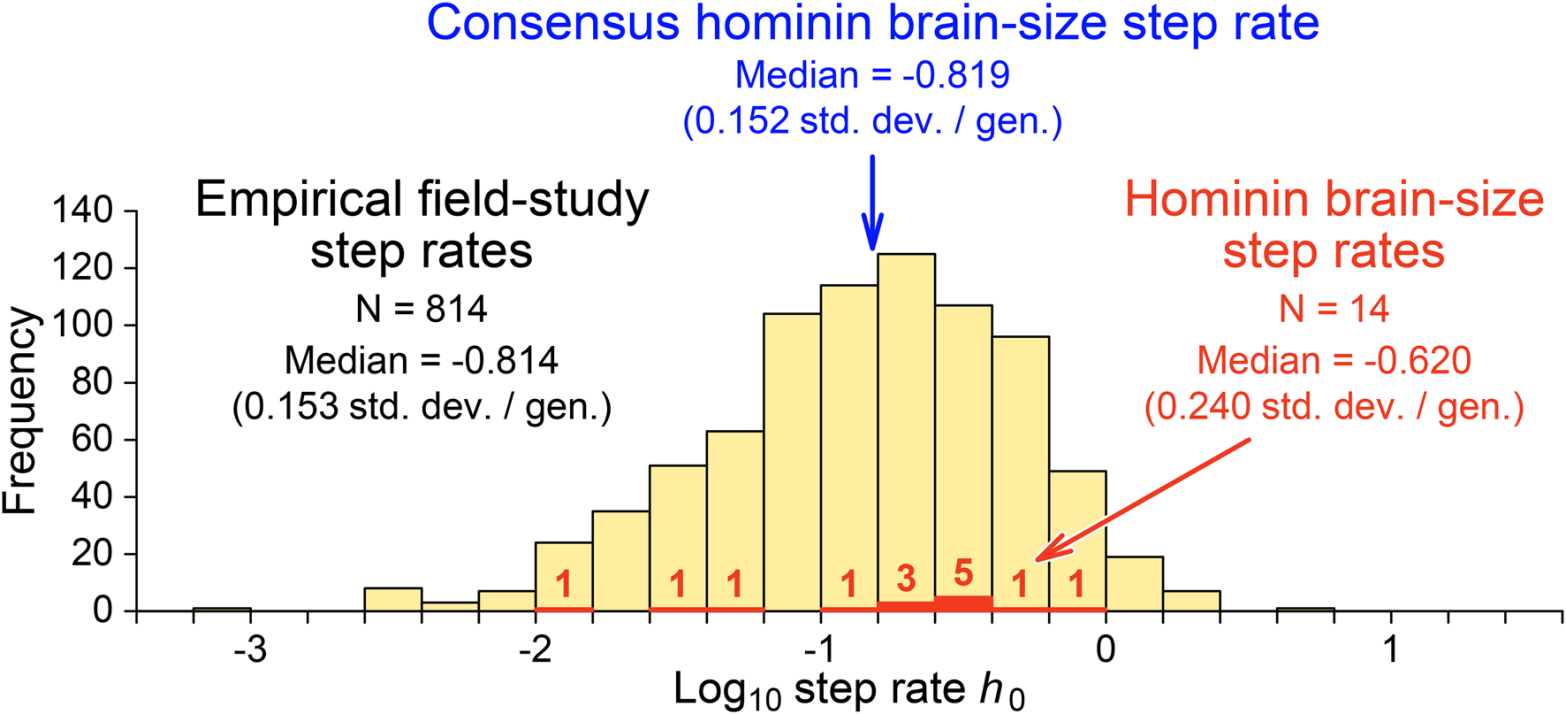
Hominin brain-size step rates for the 14 case studies analyzed here and summarized in Supplementary Table 2 (red), compared to a large sample of modern biological field-study step rates (background histogram^17^). Step rates are calibrated in phenotypic standard deviations per generation on the generation-to-generation time scale of the evolutionary process. The median log_10_ step rate of − 0.620 (*h*_0_ = 0.240) for hominin case studies is slightly higher than the median log_10_ step rate of − 0.814 (*h*_0_ = 0.15) documented in modern biological field studies. The consensus hominin brain-size step rate of − 0.819 (*h*_0_ = 0.152; Fig. 1c), in blue, is almost exactly the median documented in modern field studies.

The median intercept in Fig. 1b is − 0.819, which, exponentiated, corresponds to a step difference *h*_0_ = 10^−0.819^ = 0.152 standard deviations on a time scale of one generation. This median intercept and its corresponding step difference are almost exactly the median intercept and step difference or rate for the 814 empirical field-study step rates graphed in Fig. 2.

All of the possible rates of change between sample mean ECVs in the time series of Fig. 1a (in standard deviations per generation) are plotted against their corresponding intervals (generations) on the log-rate-interval or LRI graph of Fig. 1c. The 4747 log rates range in value from − 6.763 to − 0.879, on log intervals that again range from 1.301 to 5.106. The distribution of rates has a median slope of − 0.712. Asterisks at range limits indicate statistical significance at *p* < 0.05. The slope of − 0.712 in Fig. 1c is significantly different from the slope of 0.000 expected for directional change and from − 0.500 expected for random change (two asterisks); − 0.712 is also significantly different from the slope of − 1.000 expected for stasis (single asterisk).

The median intercept is again − 0.819, which, exponentiated, corresponds to a step rate h_0_ = 10^−0.819^ = 0.152 standard deviations per generation on a time scale of one generation. As before, this median intercept and its corresponding step rate for temporal scaling of hominin brain-size are almost exactly the median intercept and step difference or rate for the 814 empirical biology field-study step rates graphed in Fig. 2. These high rates on short-term generation-to-generation scales of time indicate the extraordinary potential for change inherent in the evolutionary process.

### Binned consensus time series

**Figure 3:**
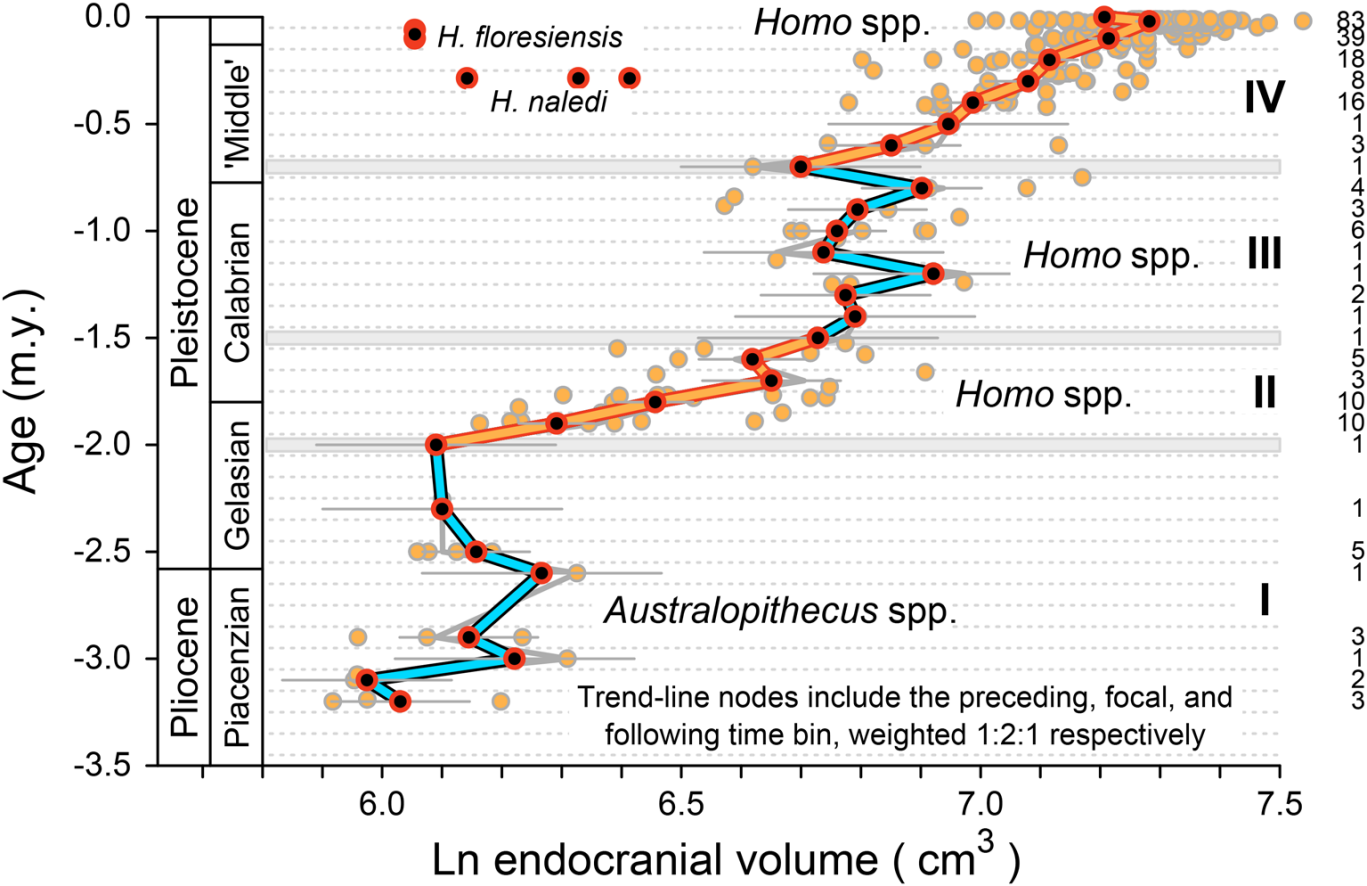
Brain size evolution in the broad lineage of Plio-Pleistocene hominins leading to modern humans, aggregated on a time scale of 0.1 million years (100 k.y.). Points in the background are the 233 hominin ECV values representing 98 geological ages shown in Fig. 1 (Supplementary Dataset 1). Gray zigzag line in the background traces mean ln ECV values for each 100 k.y. time bin through 28 successive bins. Blue and yellow lines in the foreground trace a 1:2:1-weighted running mean of ln ECV values for the 28 time bins (plus a modern sample). Sample sizes for each bin are listed in the right-hand column. Four phases of change are evident: I, in blue, an initial phase of relatively stationary brain size; II, in yellow, an early phase of increasing brain size; III, in blue, a second phase of relatively stationary brain size; and IV, in yellow, a second phase of increasing brain size. Phases are compared statistically in Fig. 4.

The time series in Fig. 1a can be simplified by binning, with 100 k.y. time bins chosen to approximate the precision typical for specimen ages. The ECV value in a time bin is the mean of ln ECV values for the interval. There are 33 100-k.y. time bins spanning the consensus Plio-Pleistocene history of hominins, and 28 of these include at least one ECV value. The binned time series is shown as the gray zig-zag line in the background of Fig. 3. Here again the independent variable, geological age (m.y.), is on the vertical axis, and the dependent variable, ln ECV (cm^3^), is on the horizontal axis. Regression of ln ECV on age yields a long-term slope or rate of ln ECV (cm^3^)/age (m.y.) = 0.379 (compared to 0.416 without binning). The intercept of ln ECV = 7.189 (compared to 7.253 without binning) corresponds to an ECV of e^7.189^ = 1325 cm^3^.

### Binned and smoothed consensus time series

The binned time series in Fig. 3 can be smoothed slightly by calculating a running average. Each node in the smoothed series is the weighted mean of preceding, focal, and succeeding time bins—weighted 1:2:1, respectively. Colored line segments in Fig. 3 show the resulting pattern of change through time. The binned and smoothed time series differs little from the underlying binned series that connects successive means without smoothing, which means smoothing has a relatively small effect.

The binned and the binned-and-smoothed time series in Fig. 3 show four phases of relative stasis and change. Phase I, which spans 13 time bins ranging in age from − 3.2 to − 2.0 m.y. before present, is a long stationary phase with little, if any, net change in ECV value. Phase II, which spans six time bins ranging in age from − 2.0 to − 1.5 m.y., is an interval of increasing brain size. Phase III, which spans nine time bins ranging in age from − 1.5 to − 0.7 m.y., is a second stationary phase. Finally, Phase IV, which spans eight time bins ranging in age from − 0.7 m.y. to − 10 k.y., is a second slightly longer interval of increasing brain size. The overall pattern ends with a slight reduction in brain size from the end of the Pleistocene to the present. The change or inflection between phases III and IV was independently identified by Ruff et al.^10^, and those between phases I and II and between phases II and III were identified by DeSilva et al.^18^. Small-brained middle Pleistocene *Homo naledi*^19,20^ and late Pleistocene *H. floresiensis*^21^ are conspicuous outliers distinct from the broad lineage leading to modern large-brained humans.

### Statistical evaluation of stasis and change

As noted, there appear to be four phases of stasis and directional change in the *Australopithecus*–*Homo* time-series illustrated in Fig. 3. These phases and the time-series segments they represent can be identified more rigorously by evaluating the statistical significance of all successive subsamples of the whole binned-and-smoothed time series—analyzing the subsamples in windows that represent the full range of starting positions, combined in turn with the full range of segment lengths. Critical values are given by the bootstrapped 95% confidence interval for the temporal scaling slope of the scatter of rates and corresponding intervals, as shown, for example, in the LRI plot of Fig. 1c.

When the minimum value of the confidence interval for a temporal scaling slope excludes − 0.5 then it also excludes − 1: the time series is significantly different from both random change and stasis, and it is hence more directional than random or stationary. When the maximum value of the confidence interval for the temporal scaling slope excludes − 0.5 then it also excludes 0: the time series is significantly different from random and directional change, and it is hence more stationary than random or directional. Time-series segments where random change cannot be excluded are considered random.

**Figure 4:**
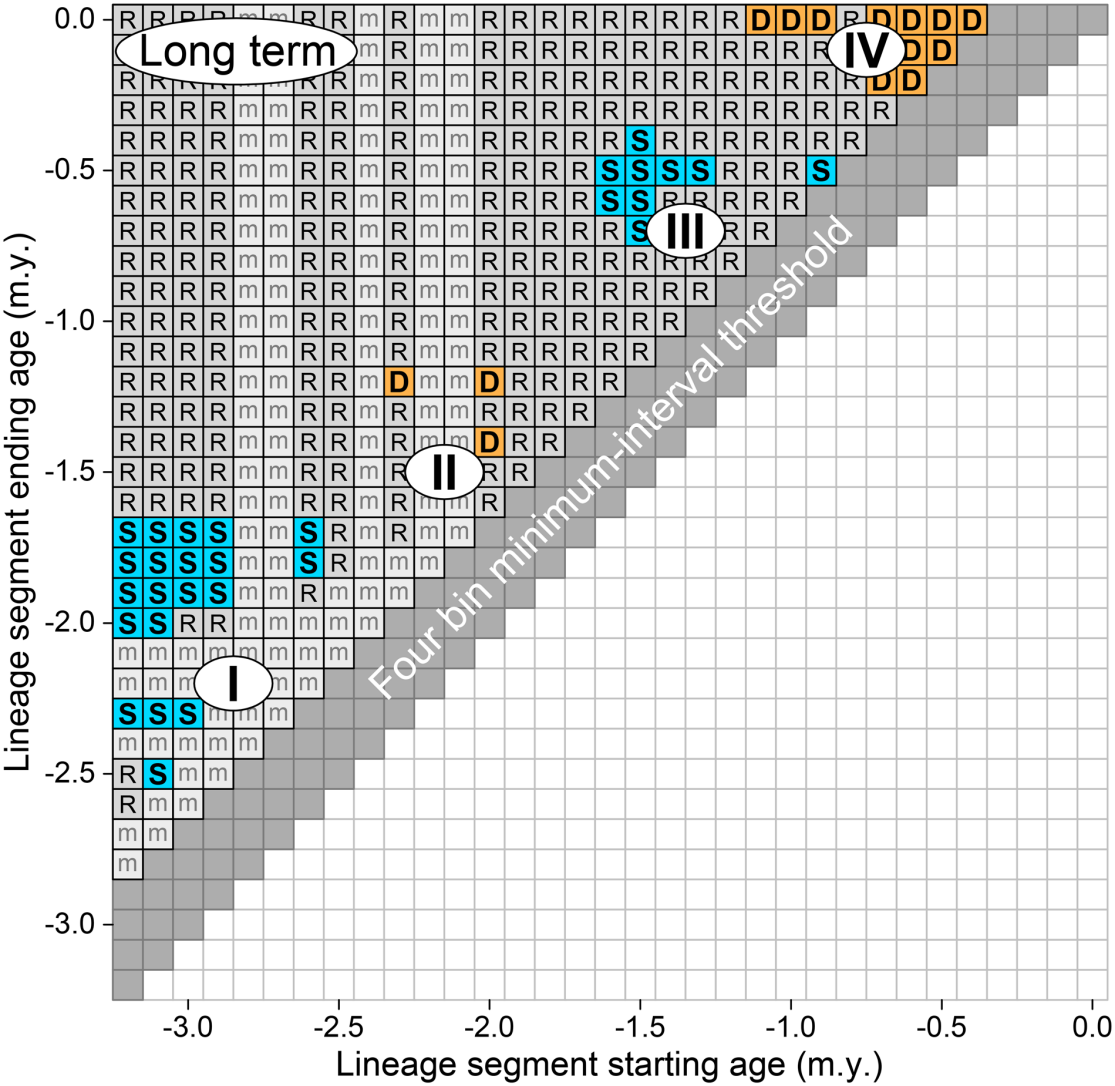
Statistical evaluation of brain size evolution in the broad lineage of Plio-Pleistocene hominins leading to modern humans (Fig. 3). The starting age for each lineage segment is shown on the abscissa and the ending age is shown on the ordinate. Empty bin segments are marked ‘m’ for missing. Each segment was analyzed following the protocol illustrated in Fig. 1c (minimum segment length required for statistical significance is five 100-k.y. bins). Time series segments with LRI temporal scaling slopes that cannot be distinguished from − 0.500 expected for random change are shown in gray and labeled ‘R’. Segments that can be distinguished from random change and directional change, representing relative stasis, are shown in blue and labeled ‘S’. Segments that can be distinguished from stasis and random change, representing directional change, are shown in yellow and labeled ‘D’. Note that the four phases identified in Fig. 3, phases I through IV, emerge as distinct modes in clusters of difference from random (again labeled I, II, etc.).

A half-matrix showing the organization and pattern of this significance testing is illustrated in Fig. 4. Construction of Fig. 4 started with the mean ln ECV value for the oldest bin of Fig. 3, the bin with a starting age of − 3.2 m.y. Statistical significance requires a minimum of five samples. The time series segment starting at − 3.2 m.y. in Fig. 3 and spanning a minimum of five samples includes the mean ln ECV value for bin 1 at a starting age of − 3.2 m.y. and those for bins 2 through 4 with starting ages of − 3.2 and ending ages of − 3.1, − 3.0, and − 2.9 m.y., respectively. There are no ECV values in the bins starting at − 3.2 and ending at − 2.8 and − 2.7 m.y., so the fifth and final ln ECV value in the initial segment is that starting at − 3.2 and ending at − 2.6 m.y. Analysis of this initial segment, following the protocol illustrated in Fig. 1c, shows that it cannot be distinguished from a time series with ln ECV changing randomly, hence the ‘*R*’ for ‘random’ entered in the Fig. 4 cell at coordinates (starting age, ending age) or (x, y) = (− 3.2, − 2.6).

The first time series segment in Fig. 3 with six values in sequential bins is that starting at − 3.2 m.y. and ending at − 2.5 m.y. This too cannot be distinguished from random, hence the ‘*R*’ entered in the Fig. 4 cell at coordinates (− 3.2, − 2.5). The first time series segment in Fig. 3 with seven values in sequential bins is that starting at − 3.2 m.y. and ending at − 2.3 m.y. This 7-step time series has a temporal scaling slope significantly different from expectation for both random and directional time series, but not significantly different from expectation for a stationary time series; hence the ‘*S*’ for ‘stationary’ entered in the corresponding cell (− 3.2, − 2.3) of Fig. 4.

Time series segments starting at − 3.2 m.y. in Fig. 3 can be extended by adding successively younger and younger bins until the final bin in the time series is reached, with a bin interval ending at 0.0 m.y. (rounded to 0.0 m.y. from − 10 k.y. =  − 0.01 m.y.). This is the entry for the full time series. The same procedure can be repeated for bins in Fig. 3 starting at − 3.1 m.y., bins starting at − 3.0 m.y., etc., until the half-matrix of Fig. 4 is filled. Cells in Fig. 4 that represent bins with missing ECV values are marked ‘*m*’ for ‘missing’ to indicate that the values are unknown.

The half-matrix of Fig. 4 is a time series map enabling stationary and directional segments to be distinguished from each other, and both to be distinguished from background random change. The map confirms that the four phases of relative stasis and change labeled with Roman numerals in the background of Fig. 3 are real and statistically significant. Phase I of Fig. 3 corresponds to the initial cluster of cells labeled ‘S’ in the lower left-hand portion of Fig. 4. Phase II corresponds to the smaller cluster of cells labeled ‘D’ that starts and ends later in time. Phase III corresponds to the second cluster of cells labeled ‘S’, and Phase IV corresponds to the cluster of cells labeled ‘D’ in the upper right-hand portion of Fig. 4. Cells representing phases I through IV in Fig. 4 are color-coded to match corresponding time series segments in Fig. 3.

### Visualization of stasis and change

There is some ambiguity concerning the boundaries between the successive stationary and directional phases I, II, III, and IV. This was first evident when attempting to reconcile placement of the boundaries in Figs. 1 and 3. The ambiguity is also evident in Fig. 4, where the earliest starting age for significant Phase II directional change at − 2.3 m.y. precedes the latest ending age for significant Phase I stasis at − 1.7 m.y. The earliest starting age for significant Phase III stasis at − 1.6 m.y., precedes the latest ending age for significant Phase II directional change at − 1.2 m.y., and the earliest starting age for significant Phase IV directional change at − 1.1 m.y., precedes the latest ending age for significant Phase III stasis at − 0.4 m.y.

**Figure 5:**
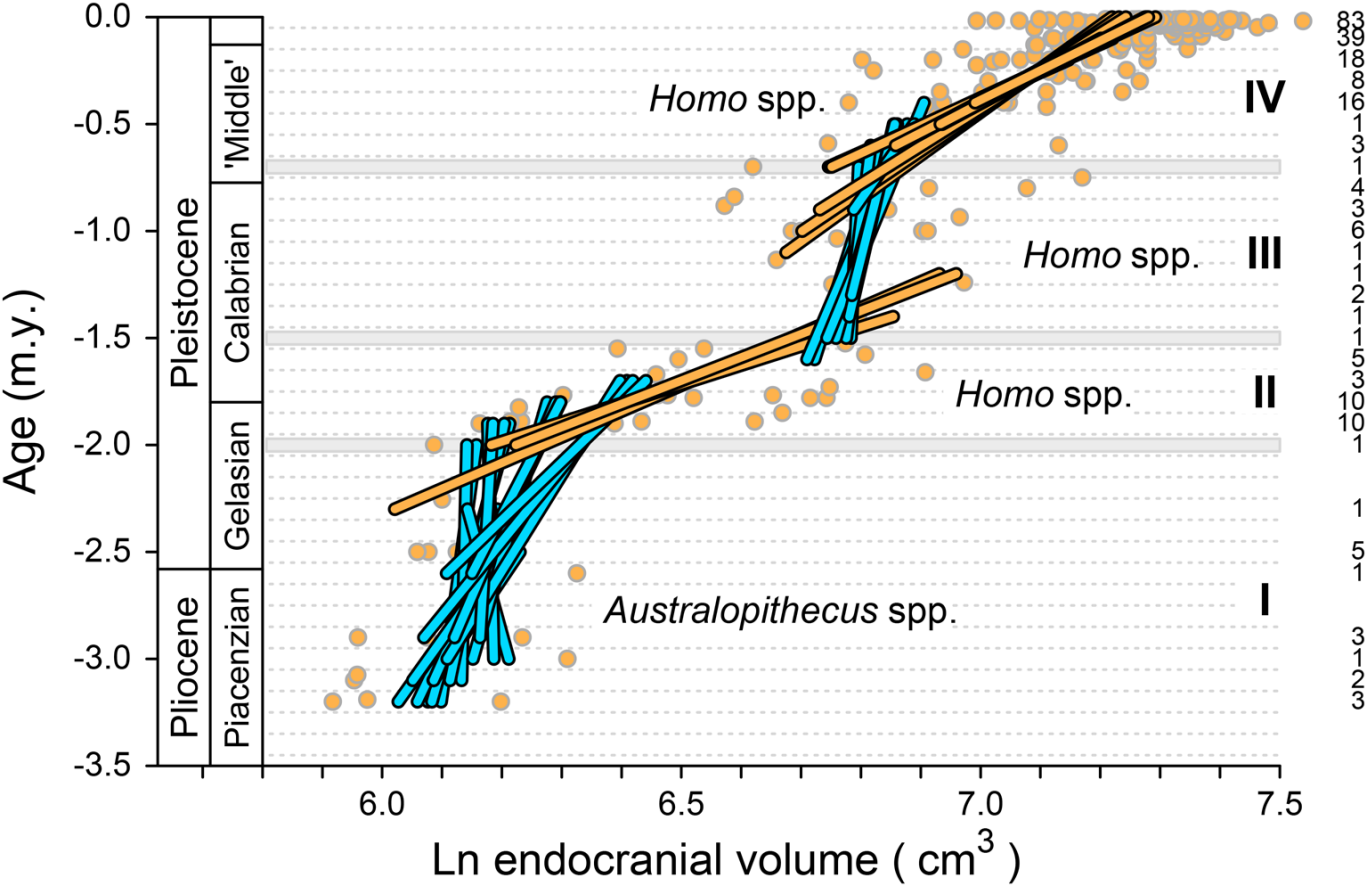
Graphical representation of the time series segments of Fig. 4 that are significantly different from random, plotted on the ln ECV and age axes of Fig. 3. Note that the Phase I–II, Phase II–III and Phase III-IV boundaries are inflections between stationary and directional time series segments, but the phase changes are diffuse in the sense that there is temporal overlap of stationary and directional segments in each transition.

The clusters of stasis (S) and directional change (D) for hominin brain size appear to be discrete on the map of Fig. 4, but there is some overlap in time between the clusters labeled I, II, III, and IV. The overlap is displayed in the bivariate graph of Fig. 5, which now includes the geological time scale of Fig. 3. Regression lines for ln ECV on age plotted in Fig. 5 are color coded to match the intervals of stasis and directional change in Fig. 4 that differ significantly from random. The successive phases in Fig. 5 labeled I, II, III, and IV are real in that they are based on stationary time series intervals statistically different from random and directional change, or based on directional time series intervals statistically different from stasis and random change. In addition, Fig. 5 shows that the phases overlap in time, meaning that the boundaries between the phases are diffuse.

### Inverse relationship of temporal scaling intercepts and slopes

**Figure 6:**
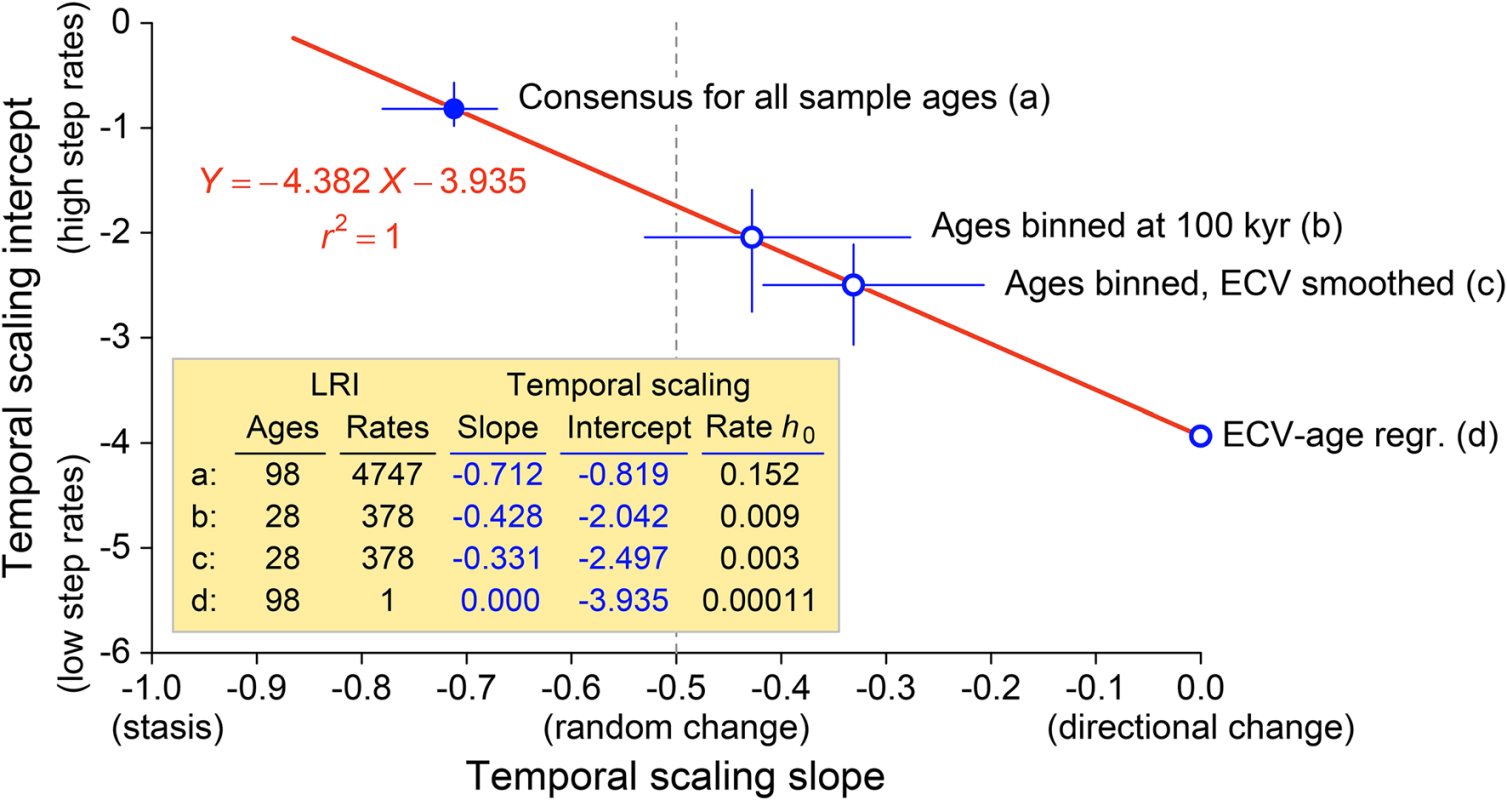
Temporal scaling slopes and intercepts for evolutionary rates of change in hominin endocranial volume analyzed in Figs. 1 and 3. (**a**) Temporal scaling of unbinned ECVs for all 98 specimen ages studied here (slope and intercept from Fig. 1c; *h*_0_ = 10^−0.819^ = 0.152). (**b**) Temporal scaling of ECVs binned by age (gray line linking bin means in the background of Fig. 3; *h*_0_ = 10^−2.042^ = 0.009). (**c**) Temporal scaling of ECVs binned by age and smoothed with a 1:2:1-weighted running mean (blue and yellow lines linking bin means in the foreground of Fig. 3; *h*_0_ = 10^−2.497^ = 0.003). (**d**) Linear regression of 233 ECV values on 98 geological ages (dashed line in Fig. 1a; *h*_0_ = 10^−3.935^ = 0.00011). Progressive filtering of higher-frequency components from the time series of Fig. 1a moves the temporal scaling slope from − 0.712 (random with a stationary component) to 0.000 (purely directional), and simultaneously reduces the temporal scaling intercept, minimizing the inferred step rate *h*_0_. Error bars for *a*, *b*, and *c* are bootstrapped 95% confidence intervals.

Figure 6 is a graph of median temporal scaling intercepts plotted against their corresponding temporal scaling slopes, showing an inverse relationship. The slopes and intercepts are taken from Fig. 1c, from parallel analyses described above for binned and binned-and-smoothed versions of the Fig. 1a time series, and from the regression in Fig. 1a. A coefficient of determination of *r*^2^ = 1 for the relationship in Fig. 6 means that the temporal scaling intercept is fully dependent on the temporal scaling slope (and vice versa). The scatter of points in Fig. 1c (where the number of levels is *n* = 98 and the number of rates is *N* = 4,747) has the same location as the scatters of points for the analyses of binned and binned-and-smoothed time series (where n = 28 and *N* = 378). Reduction of all points in Fig. 1a to a single regression yields a single rate and 0.0 as a temporal scaling slope. Figure 6 shows how altering a temporal scaling slope changes interpretation of the mode of change, and simultaneously alters interpretation of the step rates involved.

## Discussion

There are many ways to calculate and compare rates in the evolution of hominin brain size. Here rates, calibrated in phenotypic standard deviations per generation, were calculated four ways:

- tracing and comparing all sample pairs through time with no time binning or ECV smoothing (yellow trace in Fig. 1a), which yielded 4747 rates;
- tracing and comparing sample pairs in successive 100 k.y. time bins with no ECV smoothing (gray trace in the background of Fig. 3), which yielded 378 rates;
- tracing and comparing sample pairs in successive 100 k.y. time bins with 1:2:1 smoothing of ECV values (colored trace in the foreground of Fig. 3), which yielded 378 rates; and
- calculating one linear regression of all ECV values and ages (dashed line in Fig. 1a), which yielded a single rate.

The latter represents, in effect, binning and smoothing of samples and ages as if all represent one long interval. Different time scales are involved in these analyses, with the shortest time scales predominating when no binning is involved. Binning yields results on an intermediate time scale, and regression over a whole sample yields results on the longest possible time scale.

Binning ages and smoothing ECV values, alone or together, move temporal scaling slopes toward higher values, yielding an interpretation of more-directional change. Similarly, binning and smoothing, alone or together, move temporal scaling intercepts toward lower values, yielding lower step rates. Figure 6 shows the effect that lengthening the time scale has on temporal scaling intercepts and slopes when moving from (a) a simple consensus of ECV values and ages, to (b and c) binning on an intermediate time scale, to (d) a single linear regression of ECV on age over the entire interval.

The time series in the foreground of Fig. 3 is the original time series of Fig. 1a, binned and smoothed in 33 successive 100 k.y. time bins, with 1:2:1 ECV smoothing. Analysis yields the four-phase interpretation of stasis → directional change → stasis → directional change shown in Figs. 4 and 5. Analysis of all pairwise combinations of samples in the full consensus time series (Fig. 1a), with no binning or smoothing, yields high step rates on short time scales and an interpretation dominated by stasis (blue squares in Supplementary Fig. S15). A similar but slightly coarser-scale analysis of change for 65 successive 50 k.y. time bins shifts the interpretation to four phases of stasis → random change → stasis → random change (blue and gray squares in Supplementary Fig. S17). A much coarser-scale analysis of change for 17 successive 200 k.y. time bins shifts the interpretation to four phases of random change → directional change → random change → directional change (gray and yellow squares in Supplementary Fig. S19). Four phases are present in each of these interpretations, but finer scale analyses are more stationary and coarser scale analyses are more directional. Finally, fitting a linear regression model to the time series of Fig. 1a as a whole is the coarsest form of analysis. Regression yields a time-averaged rate on the time scale of the entire series, and any change present is likely to appear directional on that long time scale.

Analyses here are all based on the same underlying empirical ECV values and ages. However, the time scale of an analysis makes a difference in how we interpret both the evolutionary tempo and the evolutionary mode. Analyzed on the finest time scale, the scale of consensus sample ages, hominin brain size appears to evolve at high rates, and the evolutionary mode lies in the range of stasis and random change. Analyzed on the coarsest time scale, lumping all samples in a single linear regression, hominin brain size appears to evolve at much lower rates and the evolutionary mode is directional change. The time scale advocated here, the time scale of 100 k.y. illustrated in Figs. 3, 4 and 5, was chosen because it approximates the temporal precision of specimen ages. It is intermediate between the finest and coarsest possibilities, and it yields an interpretation of alternating stasis and directional change. Most interpretations, regardless of the modes involved, have inflection points at or near 2.0, 1.5, and 0.7 m.y. before present (Fig. 5).

The geological record of Plio-Pleistocene environmental change, the paleontological record of contemporary faunal evolution, and the anthropological record of hominin behavioral change provide no simple explanation for any of the inflection points and mode changes identified here, let alone the observed pattern of alternating modes. Hopefully clarifying the complexity of change in hominin brain size will focus attention on the problem and lead, eventually, to a better understanding of the costs and benefits of larger brains in our own evolutionary lineage. Small brains retained by the small species *Homo naledi* and *H. florisiensis* show that other paths were possible.

## Conclusions

A pattern of four phases is evident in the Plio-Pleistocene evolution of modern human brain size. Aggregation on a time scale of 100 k.y.—chosen to reflect the temporal precision of geological ages assigned to fossil specimens—yields successive phases of stasis, directional change, stasis, and directional change. Analysis on finer scales of time moves all phases closer to stasis, and analysis on coarser scales of time moves all phases toward directional change. Interpretation of a phase in terms of mode (stasis, random, or directional change) depends on the time scale chosen for analysis. Inferred rates of change are also sensitive to the time scale of analysis.

## Methods

### Specimens and taxonomy

Fourteen Pliocene-to-Recent studies^1–14^ that document the evolution of brain size in gracile hominins were combined in a consensus time series (Supplementary Dataset 1). There is substantial change in the consensus time series, and several species are represented, however no attempt was made to identify contemporary or sequential species within the Plio-Pleistocene lineage leading to modern humans. There is disagreement about the number of species involved and the species identification of some specimens. There are no conspicuous outliers in the consensus series that exceed the variability expected for contemporaneous representatives of a lineage. When a consensus emerges on phylogenetic relationships and when more specimens are available from more geographic locations—to augment statistical power—then it will be possible to extend this analysis of pattern and rate to a more complex phylogenetic and geographic history.

Some datasets combined here include megadont specimens in the genus *Australopithecus* or *Paranthropus* with species *A.* or *P. aethiopicus*, *boisei*, *garhi*, or *robustus*: these were removed before analysis because they clearly represent an independent lineage or clade. Endocranial volumes labeled ‘infant,’ ‘child,’ ‘juvenile,’ or ‘uncorrected’ were also removed. Inclusion of *A. sediba* specimen MH1 (ECV 420) at − 1.98 m.y. would change analyses slightly but not affect the conclusions. The endocranial volumes of middle Pleistocene *Homo naledi*^19,20^ and late Pleistocene *Homo floresiensis*^21^ are distinct from all species in the Plio-Pleistocene lineage leading to modern humans. *H. naledi* and *H. floresiensis* are plotted in Fig. 3, but not included in any analyses.

There are several numbering systems for Ngandong or Solo specimens, and the synthesis of Huffman et al.^22^ was used to combine these.

### Proportion and logarithms

The important difference between similar brains is not their difference in absolute size expressed on an arithmetic scale, but rather their difference in size as a proportion. This is the basis of brain allometry employed by Dubois^23^ and most subsequent authors including Jerison^24,25^. Logarithms are the most efficient way to convert a geometric scale of proportional difference to an arithmetic scale for analysis.

### Variability of human brain size

Empirically, the arithmetic mean brain weight for 906 male and female modern humans studied by Bischoff^26^ is *x̅* = 1,306 g, with a standard deviation *s* = 130 g and a coefficient of variation *s* / *x̅* = 0.10. Brain tissue has a density of 1.05 g/cm^3 ^ (ref.^27^). The Bischoff measurements expressed as endocranial volumes have a mean *x̅* = 1,244 cm^3^, standard deviation *s* = 123.8 cm^3^, and coefficient of variation *s*/*x̅* = 0.10. On a natural-logarithmic scale (base e; abbreviated ‘ln’) the mean ECV for the Bischoff sample is *x̅* = 7.13 and the standard deviation *s* = 0.10. The arithmetic mean brain weight for 1656 male and female modern humans studied by Gjukić^28^ is *x̅* = 1,286 g, with a standard deviation *s* = 117.2 g and a coefficient of variation *s*/*x̅* = 0.09. Making the same conversion for the Gjukić sample, the mean ECV on a natural-log scale is *x̅* = 7.11 and the standard deviation *s* = 0.09. Combining these, 0.10 is used to represent the standard deviation of ln brain size. The advantage of natural-log transformation is that it makes the standard deviation equivalent to the ordinary coefficient of variation^29^.

### Time, standard deviations, and rates of evolution

Time is linear, not proportional, and time in evolution is measured in generations counted on an arithmetic scale. There is no reason to use logarithms to convert a scale that is inherently arithmetic. Generation times for great apes and humans are similar, ranging from 19 to 29 years, with a median of about 25 years^30^. A generation time of 25 years is used to calculate rates of change for hominin brain size. Rates and intervals are compared on log–log proportional axes. Sensitivity tests using generation times of 20 and 30 years show that temporal scaling slopes are unaffected. Temporal scaling intercepts and inferred rates change by one to two percent.

Standard deviation units are used to measure change in morphology, following J. B. S. Haldane^15^, and the standard deviations are standard deviations after natural-log transformation. Rates are expressed in haldane units of standard deviation per generation, abbreviated *h* with a subscript^16^. The subscript is the number of generations in the rate denominator of the *h* calculation, expressed in orders of magnitude (log base 10, abbreviated ‘log’). The rate of greatest interest, the step rate *h*_0_, is the rate on a time scale of one generation (intercept on an LDI or LRI graph). This cannot be determined directly for modern humans or fossil hominins because endocranial volumes of a population have not been studied from one generation to the next. Step rates must be inferred by extrapolation from differences or rates that are calculated on multigenerational time scales (both yield the same result).

### Linear regression of brain size on time

Change in endocranial volume, like most evolutionary change, has an asymmetric relationship with time. Change in ECV requires the passage of time, but the passage of time does not require change in ECV. Time is thus an independent variable, and ECV is a dependent variable. Both are measured with error, but measurement errors are negligible compared to the structural dependency. When logged, proportional change in ECV becomes linear. Time in generations is inherently linear. Thus ordinary least-squares regression, acknowledging the dependent relationship of ln ECV on time, is the appropriate method for comparing the two and quantifying their relationship. Reduced major axis regression, a possible alternative, is not appropriate because it assumes a symmetrical relationship between the variables being compared.

### Temporal scaling of differences and rates

Temporal scaling is a way to quantify the relationship of differences between values in a time series to the intervals of time separating the values, and it is a way to quantify the relationship of rates for a time series to the time over which the rates are calculated^17^. The temporal scaling of differences and the temporal scaling of rates yield comparable information.

Differences in a time series have a power-law relationship with the intervals of time associated with the differences, meaning that the relationship is efficiently studied on a double-log ‘log-difference-interval’ or LDI graph. Similarly, rates for a time series have a power-law relationship with their associated intervals, and the relationship is again efficiently studied on a double-log ‘log-rate-interval’ or LRI graph. Logarithms to base 10 are convenient for temporal scaling because the interval lengths involved in evolutionary studies can differ by as many as six or seven orders of magnitude.

In the gracile hominins studied here, interval lengths range from about 10^2^ to 10^5^ generations. Aggregated patterns on such long time scales may not reflect the generation-to-generation dynamics of the underlying evolutionary process, so it is important to analyze change on all possible scales of time. Here, in contrast to the simple regression of ECV on time, robust linear modeling is used to find slopes and intercepts that maximize likelihood by minimizing absolute deviations from the linear model (using, e.g., the rlm script in the R ‘mass’ package^31^).

Confidence intervals for temporal scaling slopes and intercepts are bootstrapped, following Efron and Tibshirani^32^, by randomly drawing 1000 sets of the points on an LDI or LRI graph. Each set yields a slope and an intercept. When the slopes are sorted from smallest to largest, the expected value is the median, and a 95% confidence interval is the range from the 26th to 975th value. When the intercepts are sorted from smallest to largest, the expected value and confidence interval are calculated the same way.

Temporal scaling is an efficient way to compare an empirical time series to the spectrum of change possible in a time series. At one extreme, a time series can be stationary meaning that the cumulative change through time is negligible. Stationary time series have slopes that are significantly less than 0.5 on an LDI plot and significantly less than − 0.5 on an LRI plot. At the other extreme, directional time series change steadily (increasing or decreasing) through time. Directional time series have slopes significantly greater than 0.5 on an LDI plot and significantly greater than − 0.5 on an LRI plot. A random time series is a time series intermediate between stationary and directional, with an expected slope of + 0.5 on an LDI plot and − 0.5 on an LRI plot. An empirical time series can fall anywhere on the spectrum from stationary (stasis) to directional change, and the LDI and LRI slopes identified here provide salient landmarks and statistical benchmarks.

### Binning specimen ages and smoothing time-series trends

All hominid specimens with ages and ECV values are included in the consensus time series. The median difference between successive sample ages in the consensus series is 0.01 m.y. (10 k.y.). Empirically, one-sigma standard errors for hominin age estimates generally range from 5 to 8 percent of the age itself^33,34^, yielding an expected error on the order of 0.1 m.y. or 100 k.y. for ages in the middle of the hominin range. Binning samples in 0.1 m.y. time bins is effectively a low-pass filter removing higher frequency fluctuations that represent statistical noise.

Another source of noise is the volatility of sample means when sample sizes are small. Smoothing successive samples with a 1:2:1-weighted running mean alters the pattern of ln ECV change slightly, but may decrease the volatility of sample means substantially.

## Supplementary Information

Supplementary Dataset 1.

Supplementary Dataset 2.

Supplementary Information.

Supplementary Table 1.

Supplementary Table 2.

## Data Availability

All data generated or analyzed during this study are included in this published article and its supplementary information files.
