# Supplementary material for: Pattern and rate in the Plio-Pleistocene evolution of modern human brain size
Source: Sci Rep. 2022 Jul 2;12:11216. doi: 10.1038/s41598-022-15481-3 (PMC9250492; doi:10.1038/s41598-022-15481-3)
Supplement: Supplementary file 3 — Supplementary Information. [file 41598_2022_15481_MOESM3_ESM.pdf]

Pattern and rate in the Plio-Pleistocene evolution of modern human brain size

Philip D. Gingerich

Supplementary Information

Fourteen Analyses of Brain Evolution Time Series

Rates and patterns in the evolution of human brain size are analyzed for 14 time series reported in the literature. Each source provided quantitative estimates of endocranial volume (ECV) and associated geological age for multiple specimens. ECV values and ages are those reported by the authors, with mean values substituted for ln ECV (geometric mean) or geological age (arithmetic mean) within a study when either was reported as a range.

Study no. 1. Beals et al. (1984)

Beals, K. L., Smith, C. L. & Dodd, S. M. Brain size, cranial morphology, climate, and time machines [and comments and reply]. *Current Anthropology* **25**, 301-330 (1984).

Kenneth Beals and co-authors compiled information on 99 fossil hominids with endocranial volumes known at the time. Gracile forms included *Australopithecus africanus*, *Homo habilis*, *Homo erectus*, archaic *Homo sapiens/neandertalensis*, and early modern *Homo sapiens*, which came from 48 successive geological ages. A graph showing the evolution of cranial capacity was included as Beals et al.'s figure 11, where $\log_{10}$ of geological age in thousands of years was plotted on the abscissa and endocranial volume in cubic centimeters was plotted on the ordinate. Several ages and ECVs reported by Beals et al. are questionable, including those for the Taung holotype of *Australopithecus africanus*. Beals et al., estimated the age of the Taung endocast as 800 thousand years (following Partridge, 1973), and assigned it an adult cranial capacity of 440 cm^3. This combination makes the Taung specimen a conspicuous outlier.

The endocranial volumes and geological ages reported by Beals et al. (1984) are analyzed here in Figure S1a, where ln ECV (cm^3) is plotted on the abscissa and geological age (m.y.) is plotted on the ordinate. Age is the independent variable and ECV is the dependent variable. Regression of ln ECV on age yields a long-term slope or rate of ln ECV (cm^3) / age (m.y.) = 0.454. Converting to standard deviation units and generations (where the standard deviation of ln ECV = 0.100 cm^3 and one generation = 25 years), this is a long-term rate R = 4.536 std. dev. / 40,000 gen. = 0.000113 std. dev. / gen. — on a time scale or interval I = 2,490,000 / 25 = 99,600 generations. The corresponding difference is $D = R \cdot I$ = 11.294 standard deviations. The intercept of ln ECV = 7.254 (cm^3) corresponds to an ECV of 1,414 cm^3.

All of the possible differences between sample mean ECVs in the time series of Figure S1a (expressed in standard deviation units) are plotted against their corresponding intervals (expressed in generations) on the log-difference-interval or LDI graph of Figure S1b. The 1124 log differences range in value from −2.289 to 1.138, on log intervals ranging from 1.602 to 4.998. The distribution of differences has a median slope of 0.196, which is significantly different from the slopes expected for a stationary time series, random change, or purely directional change (asterisks), but 0.196 is closer to expectation for a stationary time series than

it is to expectation for a random walk. The median intercept is −0.543, which, exponentiated ($10^{-0.543}$), corresponds to a step difference of 0.286 standard deviations on a time scale of one generation.

All of the possible rates of change between sample mean ECVs in the time series of Figure S1a (in standard deviations per generation) are plotted against their corresponding intervals (generations) on the log-rate-interval or LRI graph of Figure S1c. The 1124 log rates range in value from −6.267 to −1.144, on log intervals that again range from 1.602 to 4.998. The distribution of rates has a median slope of −0.804, which is again significantly different from the slopes expected for a stationary time series, random change, or purely directional change (asterisks), but −0.804 is closer to expectation for a stationary time series than it is to expectation for a random walk. The median intercept is again −0.543, which, exponentiated, corresponds to a step rate h_0 = 0.286 standard deviations per generation on a time scale of one generation.

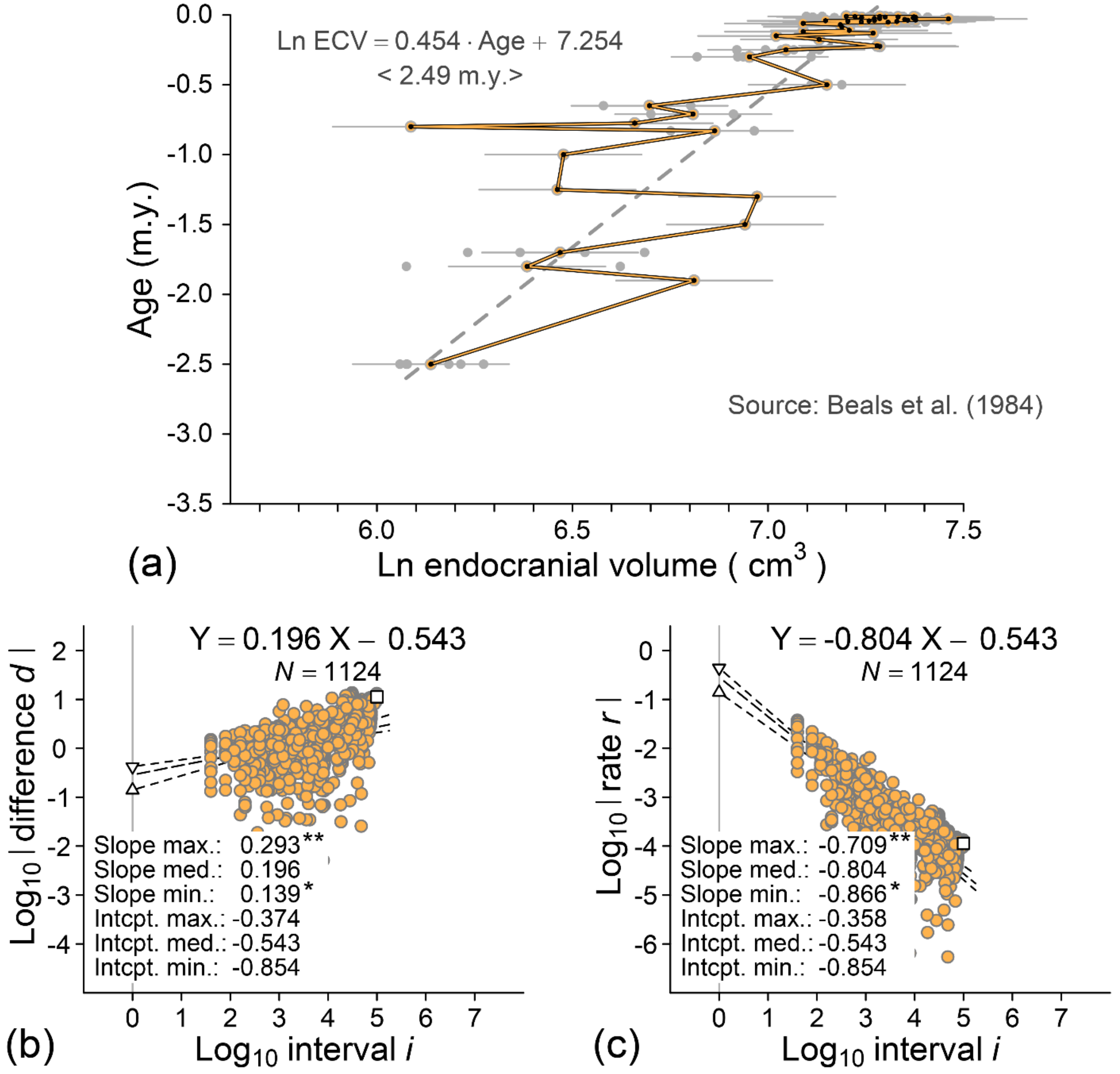

Supplementary Figure S1. Plio-Pleistocene evolution of gracile hominin endocranial volume. (a) Pattern of change through time following Beals et al. (1984). Regression of ln endocranial volume on geological age for 99 specimens yields a long term rate of 0.454 ln units per million years (ca. 1.134×10^{-4} standard deviations per generation) on a time scale of 2.490 m.y. (ca. 99,600 generations). Yellow line traces mean ECV values through geological time. (b) LDI or log difference versus interval plot of change per unit time for all combinations of differences (standard deviation units) and intervals (generations) in the time series of panel *a*. (c) LRI or log rate versus interval plot of change per unit time for all combinations of rates (standard deviations per generation) and intervals (generations) in the time series of panel *a*. Note that LDI and LRI slopes of 0.196 and −0.804 differ by one unit: each indicates a time series between expectation for stasis and random change. LDI and LRI intercepts are equal and indicate a step rate of $10^{-0.543} = 0.29$ standard deviations per generation on a time scale of one generation. The long-term difference and rate of change in panel *a* (dashed line) are plotted as open squares in panels *b* and *c*.

Study no. 2. Aiello and Dunbar (1993)

Aiello, L. C. & Dunbar, R. I. M. Neocortex size, group size, and the evolution of language. *Current Anthropology* **34**, 184-193 (1993).

Leslie Aiello and Robin Dunbar compiled information on 75 fossil hominids with endocranial volumes known at the time. Gracile forms included *Australopithecus africanus*, *Homo habilis*, *H. rudolfensis, H. erectus*, archaic *H. sapiens, H. s. neandertalensis*, early modern *H. sapiens*, and modern H. sapiens, which came from 37 successive geological ages. No graph showing the evolution of cranial capacity was included.

The endocranial volumes and geological ages reported by Aiello and Dunbar (1993) are analyzed here in Figure S2a, where ln ECV (cm^3) is plotted on the abscissa and geological age (m.y.) is plotted on the ordinate. Age is the independent variable and ECV is the dependent variable. Regression of ln ECV on age yields a long-term slope or rate of ln ECV (cm^3) / age (m.y.) = 0.421. Converting to standard deviation units and generations (where the standard deviation of ln ECV = 0.100 cm^3 and one generation = 25 years), this is a long-term rate R = 4.213 std. dev. / 40,000 gen. = 0.000105 std. dev. / gen. — on a time scale or interval I = 2,970,000 / 25 = 118,800 generations. The corresponding difference is $D = R \cdot I = 12.513$ standard deviations. The intercept of ln ECV = 7.251 (cm^3) corresponds to an ECV of 1,409 cm^3.

All of the possible differences between sample mean ECVs in the time series of Figure S2a (expressed in standard deviation units) are plotted against their corresponding intervals (expressed in generations) on the log-difference-interval or LDI graph of Figure S2b. The 666 log differences range in value from −2.155 to 1.126, on log intervals ranging from 1.903 to 5.075. The distribution of differences has a median slope of 0.399, which is significantly different from the slopes expected for a stationary time series or purely directional change (asterisks), but not from 0.500 expected for a random walk. The median intercept is −1.231, which, exponentiated ($10^{-1.231}$), corresponds to a step difference of 0.059 standard deviations on a time scale of one generation.

All of the possible rates of change between sample mean ECVs in the time series of Figure S2a (in standard deviations per generation) are plotted against their corresponding intervals (generations) on the log-rate-interval or LRI graph of Figure S2c. The 666 log rates range in value from −5.550 to −1.680, on log intervals that again range from 1.903 to 5.075. The distribution of rates has a median slope of −0.601, which is again significantly different from the slopes expected for a stationary time series or purely directional change (asterisks), but not from the slope of −0.500 expected for a random walk. The median intercept is again −1.231, which, exponentiated, corresponds to a step rate h_0 = 0.059 standard deviations per generation on a time scale of one generation.

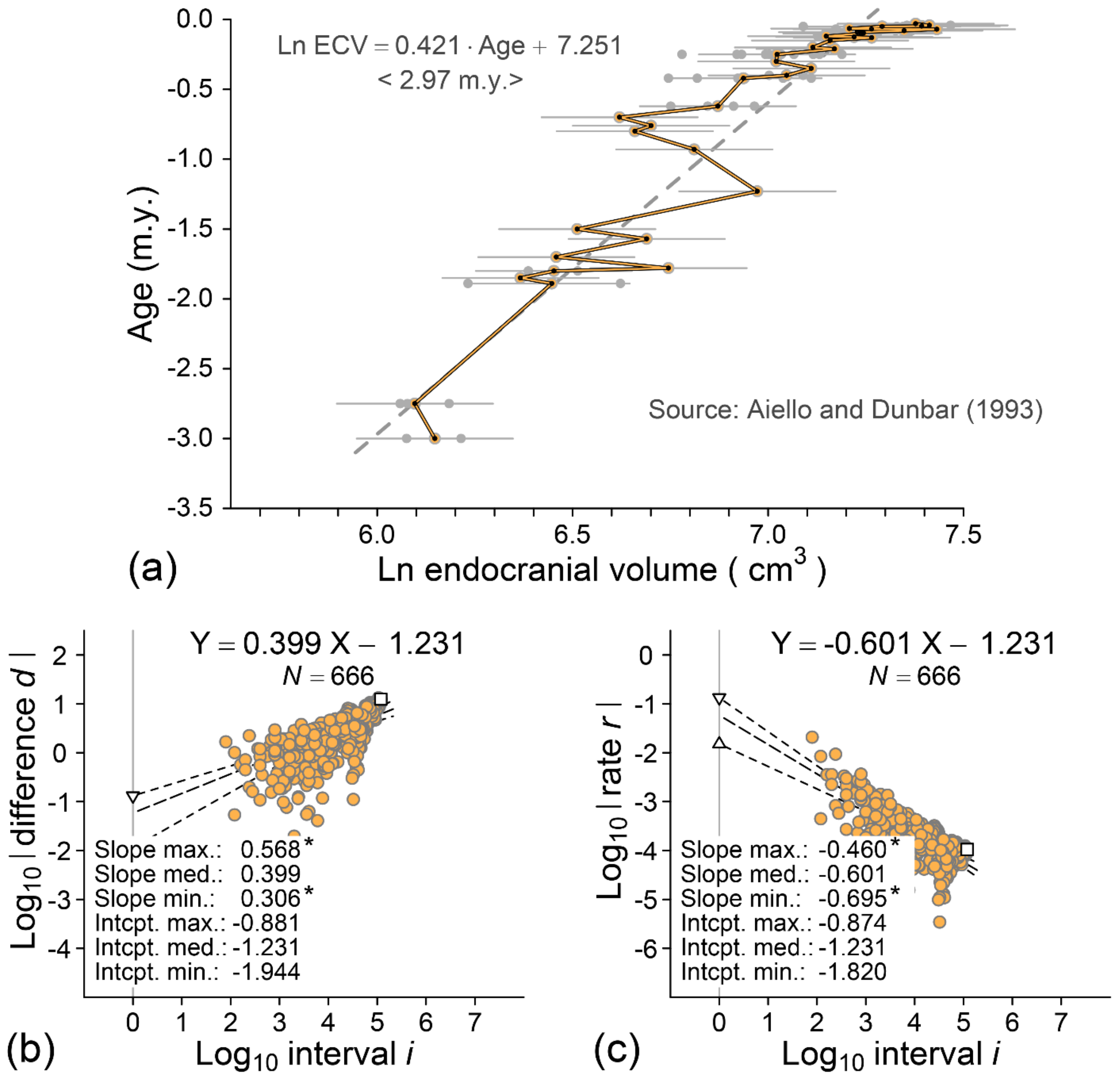

Supplementary Figure S2. Plio-Pleistocene evolution of gracile hominin endocranial volume. (a) Pattern of change through time following Aiello and Dunbar (1993). Regression of ln endocranial volume on geological age for 75 specimens yields a long term rate of 0.421 ln units per million years (ca. 1.053×10^{-4} standard deviations per generation) on a time scale of 2.970 m.y. (ca. 118,800 generations). Yellow line traces mean ECV values through geological time. (b) LDI or log difference versus interval plot of change per unit time for all combinations of differences (standard deviation units) and intervals (generations) in the time series of panel *a*. (c) LRI or log rate versus interval plot of change per unit time for all combinations of rates (standard deviations per generation) and intervals (generations) in the time series of panel *a*. Note that LDI and LRI slopes of 0.399 and −0.601 differ by one unit: neither can be distinguished from random change. LDI and LRI intercepts are equal and indicate a step rate of $10^{-1.231}$ = 0.059 standard deviations per generation on a time scale of one generation. The long-term difference and rate of change in panel *a* (dashed line) are plotted as open squares in panels *b* and *c*.

Study no. 3. Stanyon et al. (1993)

Stanyon, R., Consigliere, S. & Moreschalchi, M. A. Cranial capacity in hominid evolution. *Human Evolution* **8**, 205-216 (1993).

Roscoe Stanyon and co-authors analyzed a set of hominid crania with endocranial volumes and geological ages. This included 110 gracile hominin crania from 42 successive geological ages representing *Australopithecus afarensis*, *Australopithecus africanus*, *Homo habilis*, *Homo erectus*, archaic *Homo sapiens*, *Homo sapiens neandertalensis*, and *Homo sapiens sapiens*. These were graphed two ways, as cranial capacity versus geological age and, for *Homo* spp., as cranial capacity versus log geological age.

The endocranial volumes and geological ages reported by Stanyon et al. (1993) are analyzed in Figure S3a, where ln ECV (cm^3) is plotted on the abscissa and geological age (m.y.) is plotted on the ordinate. Age is the independent variable and ECV is the dependent variable. Regression of ln ECV on age yields a long-term slope or rate of ln ECV (cm^3) / age (m.y.) = 0.427. Converting to standard deviation units and generations (where the standard deviation of ln ECV = 0.100 cm^3 and one generation = 25 years), this is a long-term rate R = 4.271 std. dev. / 40,000 gen. = 0.000107 std. dev. / gen. — on a time scale or interval I = 3,290,000 / 25 = 131,600 generations. The corresponding difference is $D = R \cdot I$ = 14.051 standard deviations. The intercept of ln ECV = 7.245 (cm^3) corresponds to an ECV of 1,402 cm^3.

All of the possible differences between sample mean ECVs in the time series of Figure S3a (expressed in standard deviation units) are plotted against their corresponding intervals (expressed in generations) on the log-difference-interval or LDI graph of Figure S3b. The 860 log differences range in value from −1.830 to 1.131, on log intervals ranging from 2.602 to 5.119. The distribution of differences has a median slope of 0.480, which is significantly different from the slopes expected for a stationary time series or purely directional change (asterisks), but not significantly different from the slope expected for a random time series. The median intercept is −1.579, which, exponentiated ($10^{-1.579}$), corresponds to a step difference of 0.026 standard deviations on a time scale of one generation.

All of the possible rates of change between sample mean ECVs in the time series of Figure S3a (in standard deviations per generation) are plotted against their corresponding intervals (generations) on the log-rate-interval or LRI graph of Figure S3c. The 860 log rates range in value from −5.586 to −2.204, on log intervals that again range from 2.602 to 5.119. The distribution of rates has a median slope of −0.520, which is again significantly different from the slopes expected for a stationary time series or purely directional change (asterisks), but not significantly different from the slope expected for a random time series. . The median intercept is again −1.579, which, exponentiated, corresponds to a step rate h_0 = 0.026 standard deviations per generation on a time scale of one generation.

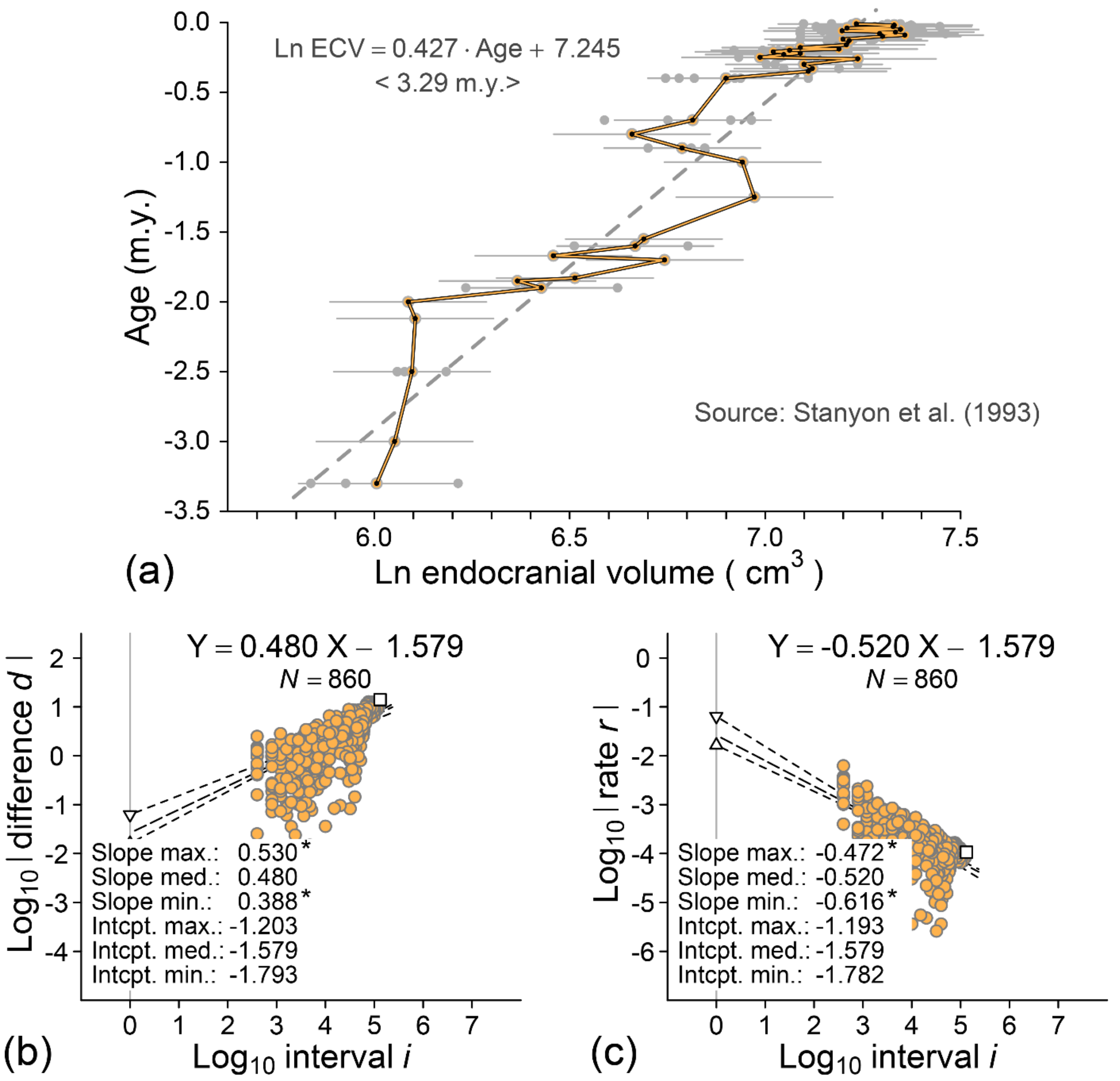

Supplementary Figure S3. Plio-Pleistocene evolution of gracile hominin endocranial volume. (a) Pattern of change through time following Stanyon et al. (1993). Regression of ln endocranial volume on geological age for 110 specimens yields a long term rate of 0.427 ln units per million years (ca. 1.068×10^{-4} standard deviations per generation) on a time scale of 3.290 m.y. (ca. 131,600 generations). Yellow line traces mean ECV values through geological time. (b) LDI plot of change per unit time for all combinations of differences (standard deviation units) and intervals (generations) in the time series of panel *a*. (c) LRI plot of change per unit time for all combinations of rates (standard deviations per generation) and intervals (generations) in the time series of panel *a*. Note that LDI and LRI slopes of 0.480 and −0.520 differ by one unit: each indicates a time series of predominantly random change. LDI and LRI intercepts are equal and indicate a step rate of $10^{-1.579} = 0.03$ standard deviations per generation on a time scale of one generation. The long-term difference and rate of change in panel *a* (dashed line) are plotted as open squares in panels *b* and *c*.

Study no. 4. Ruff et al. (1997)

Ruff, C. B., Trinkaus, E. & Holliday, T. W. Body mass and encephalization in Pleistocene *Homo*. *Nature* **387**, 173-176 (1997).

Christopher Ruff and co-authors compiled information on encephalization in 107 representatives of Pleistocene *Homo* with endocranial volumes known at the time. Specimens were grouped by temporal range, but they were not identified explicitly to species. The specimens came from 38 successive geological ages, starting at 1.8 m.y. before present. Results for cranial capacity and encephalization were tabulated, but not shown graphically.

The endocranial volumes and geological ages reported by Ruff et al. (1997) are analyzed here in Figure S4a, where ln ECV (cm^3) is plotted on the abscissa and geological age (m.y.) is plotted on the ordinate. Age is the independent variable and ECV is the dependent variable. Regression of ln ECV on age yields a long-term slope or rate of ln ECV (cm^3) / age (m.y.) = 0.386. Converting to standard deviation units and generations (where the standard deviation of ln ECV = 0.100 cm^3 and one generation = 25 years), this is a long-term rate R = 3.860 std. dev. / 40,000 gen. = 0.000097 std. dev. / gen. — on a time scale or interval I = 1,790,000 / 25 = 71,600 generations. The corresponding difference is $D = R \cdot I$ = 6.910 standard deviations. The intercept of ln ECV = 7.286 (cm^3) corresponds to an ECV of 1,460 cm^3.

All of the possible differences between sample mean ECVs in the time series of Figure S4a (expressed in standard deviation units) are plotted against their corresponding intervals (expressed in generations) on the log-difference-interval or LDI graph of Figure S4b. The 702 log differences range in value from −1.960 to 0.944, on log intervals ranging from 1.602 to 4.855. The distribution of differences has a median slope of 0.201, which is significantly different from the slopes expected for a stationary time series, random change, or purely directional change (asterisks), but 0.201 is closer to expectation for a stationary time series than it is to expectation for a random walk. The median intercept is −0.557, which, exponentiated ($10^{-0.557}$), corresponds to a step difference of 0.278 standard deviations on a time scale of one generation.

All of the possible rates of change between sample mean ECVs in the time series of Figure S4a (in standard deviations per generation) are plotted against their corresponding intervals (generations) on the log-rate-interval or LRI graph of Figure S4c. The 702 log rates range in value from −6.333 to −1.192, on log intervals that again range from 1.602 to 4.855. The distribution of rates has a median slope of −0.799, which is again significantly different from the slopes expected for a stationary time series, random change, or purely directional change (asterisks), but −0.799 is closer to expectation for a stationary time series than it is to expectation for a random walk. The median intercept is again −0.557, which, exponentiated, corresponds to a step rate h_0 = 0.278 standard deviations per generation on a time scale of one generation.

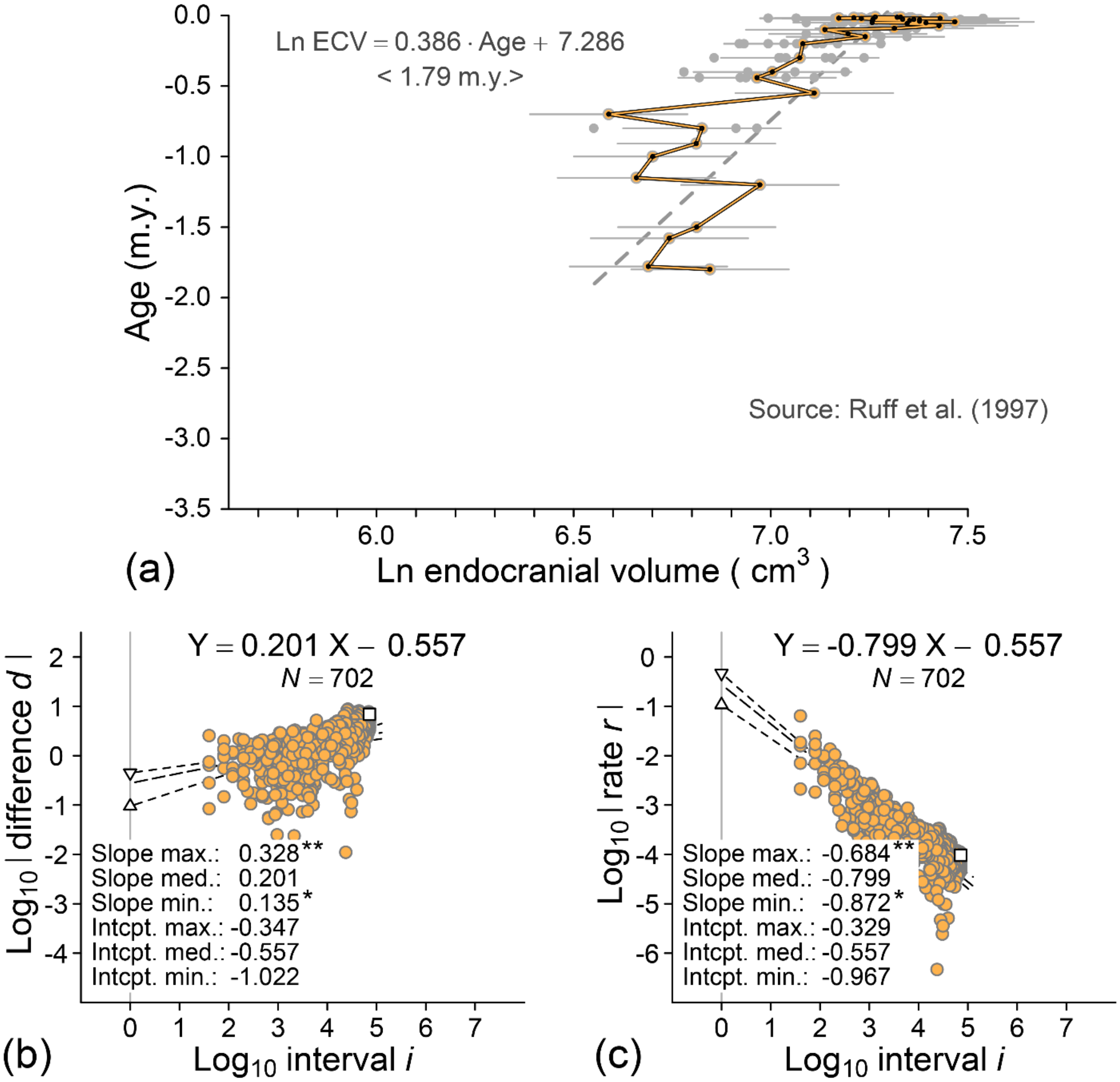

Supplementary Figure S4. Plio-Pleistocene evolution of gracile hominin endocranial volume. (a) Pattern of change through time following Ruff et al. (1997). Regression of ln endocranial volume on geological age for 107 specimens yields a long term rate of 0.387 ln units per million years (ca. 9.667×10^{-5} standard deviations per generation) on a time scale of 1.789 m.y. (ca. 71,560 generations). Yellow line traces mean ECV values through geological time. (b) LDI plot of change per unit time for all combinations of differences (standard deviation units) and intervals (generations) in the time series of panel *a*. (c) LRI plot of change per unit time for all combinations of rates (standard deviations per generation) and intervals (generations) in the time series of panel *a*. Note that LDI and LRI slopes of 0.206 and −0.794 differ by one unit: each indicates a time series between expectation for stasis and random change. LDI and LRI intercepts are equal and indicate a step rate of $10^{-0.563} = 0.27$ standard deviations per generation on a time scale of one generation. The long-term difference and rate of change in panel *a* (dashed line) are plotted as open squares in panels *b* and *c*.

Study no. 5. D'Amore et al. (2001)

D'Amore, G., Patrizio, F. & Vančata, V. Process of encephalization in hominid evolution: preliminary results of biostatistic analysis of brain size phylogenetic changes. *Anthropologie, Moravian Museum* **39**, 215-226 (2001).

Giuseppe D'Amore and co-authors compiled information on encephalization in gracile Pliocene and Pleistocene hominids. Their sample included endocranial volumes for 164 gracile hominid crania from 61 successive geological ages identified as *Australopithecus afarensis*, *A. africanus*, early *Homo*, early and late *Homo erectus*, early and late archaic *Homo sapiens*, Neanderthals, and early and late anatomically modern *Homo sapiens*. The authors calculated and graphed a novel index of encephalization compared to geological age.

The endocranial volumes and geological ages reported by D'Amore et al. (2001) are analyzed here in Figure S5a, where ln ECV (cm^3) is plotted on the abscissa and geological age (m.y.) is plotted on the ordinate. Age is the independent variable and ECV is the dependent variable. Regression of ln ECV on age yields a long-term slope or rate of ln ECV (cm^3) / age (m.y.) = 0.415. Converting to standard deviation units and generations (where the standard deviation of ln ECV = 0.100 cm^3 and one generation = 25 years), this is a long-term rate R = 4.151 std. dev. / 40,000 gen. = 0.000104 std. dev. / gen. — on a time scale or interval I = 3,190,000 / 25 = 127,600 generations. The corresponding difference is $D = R \cdot I = 13.240$ standard deviations. The intercept of ln ECV = 7.293 (cm^3) corresponds to an ECV of 1,470 cm^3.

All of the possible differences between sample mean ECVs in the time series of Figure S5a (expressed in standard deviation units) are plotted against their corresponding intervals (expressed in generations) on the log-difference-interval or LDI graph of Figure S5b. The 1,829 log differences range in value from −2.201 to 1.182, on log intervals ranging from 0.903 to 5.106. The distribution of differences has a median slope of 0.244, which is significantly different from the slopes expected for a stationary time series, random change, or purely directional change (asterisks), but 0.244 is a little closer to expectation for a stationary time series than it is to expectation for a random walk. The median intercept is −0.742, which, exponentiated ($10^{-0.742}$), corresponds to a step difference of 0.181 standard deviations on a time scale of one generation.

All of the possible rates of change between sample mean ECVs in the time series of Figure S5a (in standard deviations per generation) are plotted against their corresponding intervals (generations) on the log-rate-interval or LRI graph of Figure S5c. The 1,829 log rates range in value from −5.690 to −0.838, on log intervals that again range from 0.903 to 5.106. The distribution of rates has a median slope of −0.756, which is again significantly different from the slopes expected for a stationary time series, random change, or purely directional change (asterisks), but −0.756 is a little closer to expectation for a stationary time series than it is to expectation for a random walk. The median intercept is again −0.742, which, exponentiated, corresponds to a step rate h_0 = 0. 0.181 standard deviations per generation on a time scale of one generation.

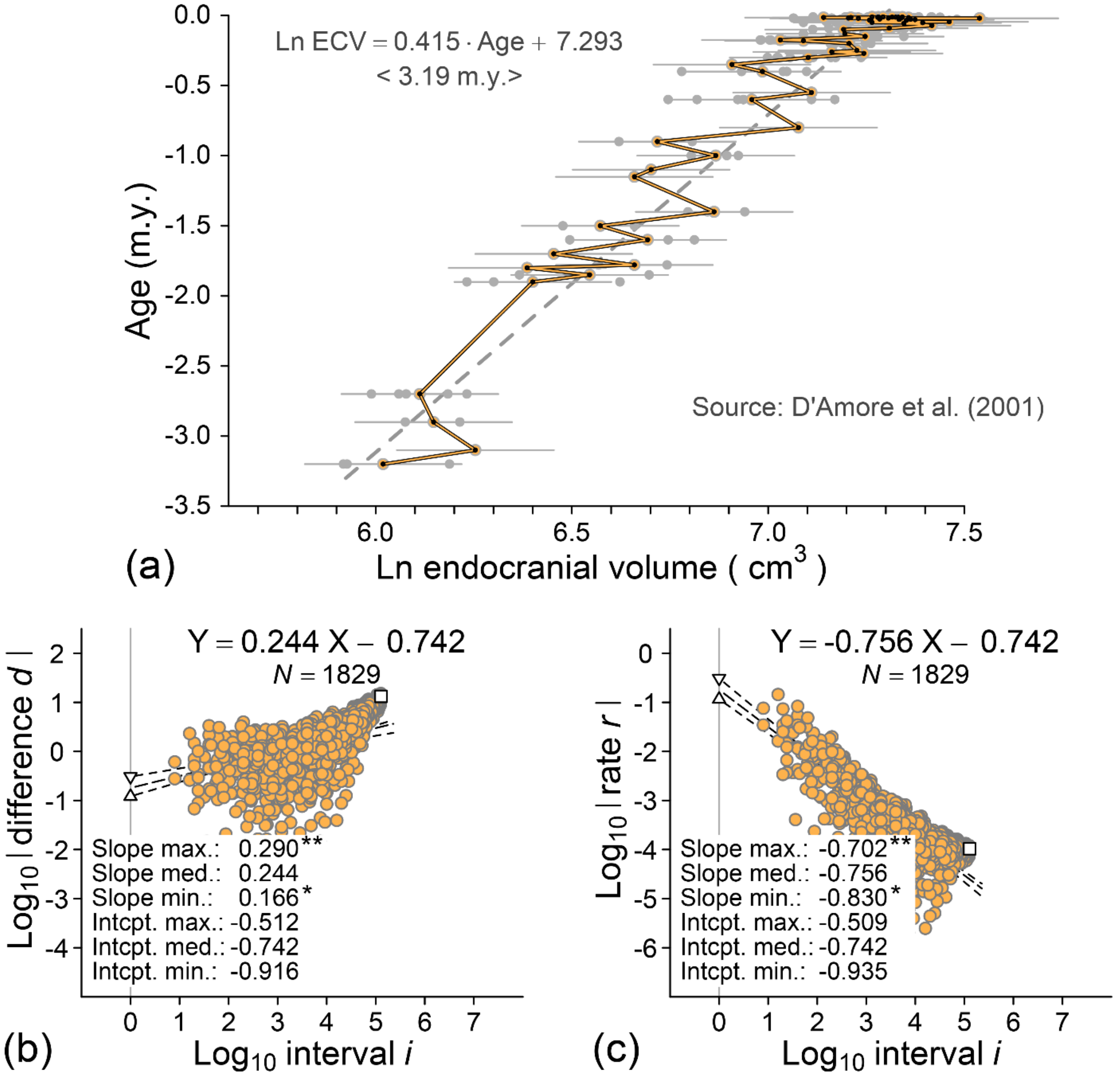

Supplementary Figure S5. Plio-Pleistocene evolution of gracile hominin endocranial volume. (a) Pattern of change through time following D'Amore et al. (2001). Regression of ln endocranial volume on geological age for 164 specimens yields a long term rate of 0.415 ln units per million years (ca. 1.037×10^{-4} standard deviations per generation) on a time scale of 3.190 m.y. (ca. 126,600 generations). Yellow line traces mean ECV values through geological time. (b) LDI plot of change per unit time for all combinations of differences (standard deviation units) and intervals (generations) in the time series of panel *a*. (c) LRI plot of change per unit time for all combinations of rates (standard deviations per generation) and intervals (generations) in the time series of panel *a*. Note that LDI and LRI slopes of 0.244 and −0.756 differ by one unit: each indicates a time series between expectation for stasis and random change. LDI and LRI intercepts are equal and indicate a step rate of $10^{-0.742} = 0.18$ standard deviations per generation on a time scale of one generation. The long-term difference and rate of change in panel *a* (dashed line) are plotted as open squares in panels *b* and *c*.

Study no. 6. Miguel and Henneberg (2001)

Henneberg, M. Hominid cranial capacity change through time: a darwinian process. *Human Evolution* **2**, 213-220 (1987).
Miguel, C. d. & Henneberg, M. Variation in hominid brain size: how much is due to method? *Homo* **52**, 3-58 (2001).

Carmen de Miguel and Maciej Henneberg compiled an exhaustive database of 606 brain size estimates for 243 fossil hominid specimens. Gracile forms, 200 specimens, included *Australopithecus afarensis*, *Australopithecus africanus*, *Homo habilis*, *Homo erectus*, archaic *Homo sapiens/neandertalensis*, and early modern *Homo sapiens*, which came from 71 successive geological ages. Miguel and Henneberg included a graph showing the evolution of cranial capacity in their figures 1 and 2, where geological ages in thousands of years were plotted on the abscissa and endocranial volumes in cubic centimeters were plotted on the ordinate. Following Henneberg (1987), Miguel and Henneberg represented the observed change in hominid endocranial volume through time, with or without robust australopithecines, as a double exponential curve.

The endocranial volumes and geological ages reported by Miguel and Henneberg (2001) are analyzed here in Figure S6a, where ln ECV (cm^3) is plotted on the abscissa and geological age (m.y.) is plotted on the ordinate. Age is the independent variable and ECV is the dependent variable. Regression of ln ECV on age yields a long-term slope or rate of ln ECV (cm^3) / age (m.y.) = 0.425. Converting to standard deviation units and generations (where the standard deviation of ln ECV = 0.100 cm^3 and one generation = 25 years), this is a long-term rate R = 4.246 std. dev. / 40,000 gen. = 0.000106 std. dev. / gen. — on a time scale or interval I = 3,190,000 / 25 = 127,600 generations. The corresponding difference is $D = R \cdot I = 13.544$ standard deviations. The intercept of ln ECV = 7.272 (cm^3) corresponds to an ECV of 1,439 cm^3.

All of the possible differences between sample mean ECVs in the time series of Figure S6a (expressed in standard deviation units) are plotted against their corresponding intervals (expressed in generations) on the log-difference-interval or LDI graph of Figure S6b. The 2,484 log differences range in value from −2.631 to 1.160, on log intervals ranging from 1.602 to 5.106. The distribution of differences has a median slope of 0.203, which is significantly different from the slopes expected for a stationary time series, random change, or purely directional change (asterisks), but 0.203 is closer to expectation for a stationary time series than it is to expectation for a random walk. The median intercept is −0.551, which, exponentiated ($10^{-0.551}$), corresponds to a step difference of 0.281 standard deviations on a time scale of one generation.

All of the possible rates of change between sample mean ECVs in the time series of Figure S6a (in standard deviations per generation) are plotted against their corresponding intervals (generations) on the log-rate-interval or LRI graph of Figure S6c. The 2,484 log rates range in value from −6.188 to −1.163, on log intervals that again range from 1.602 to 5.106. The distribution of rates has a median slope of −0.797, which is again significantly different from the slopes expected for a stationary time series, random change, or purely directional change (asterisks), but −0.797 is closer to expectation for a stationary time series than it is to expectation for a random walk. The median intercept is again −0.551, which, exponentiated, corresponds to a step rate h_0 = 0.281 standard deviations per generation on a time scale of one generation.

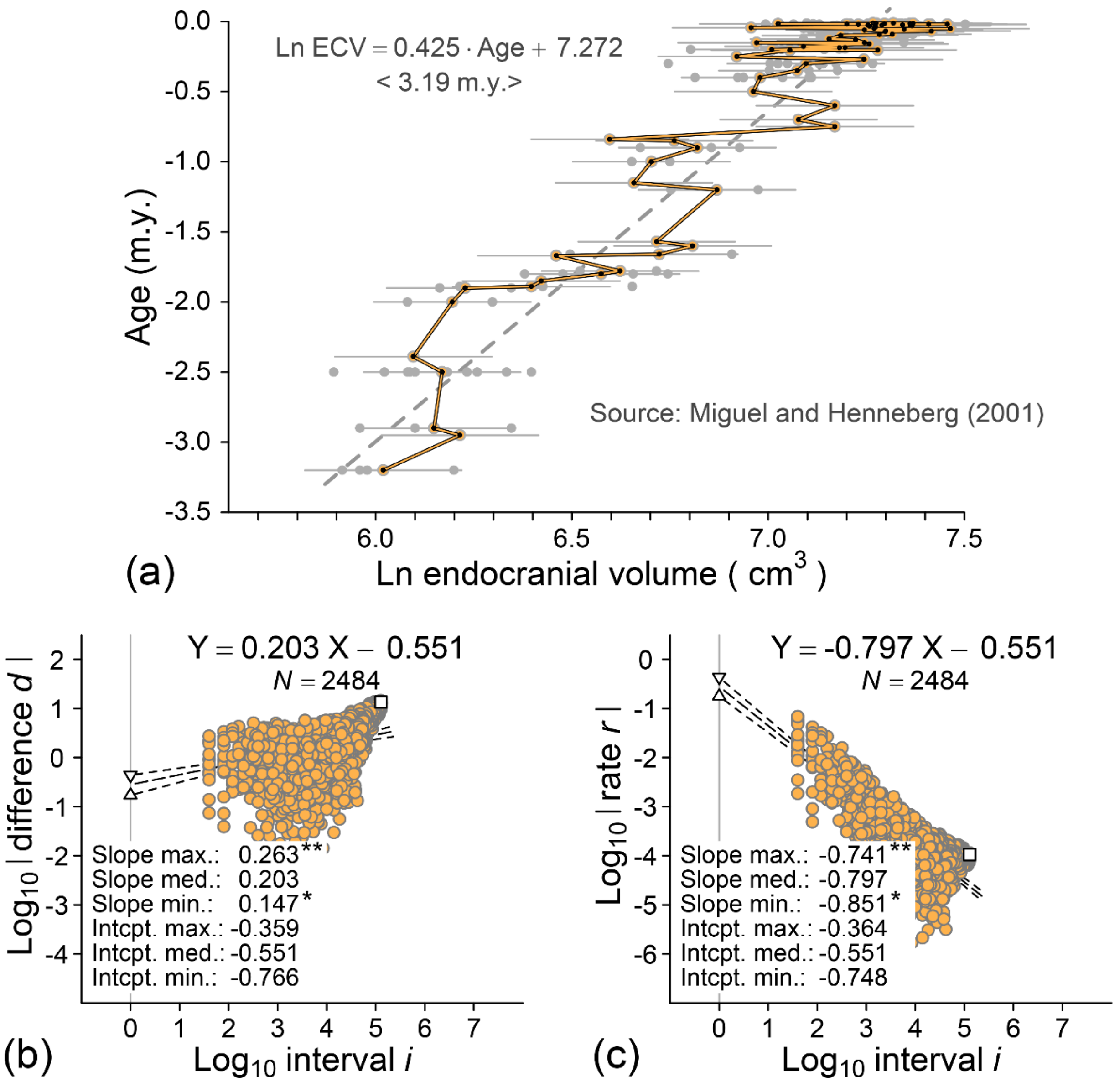

Supplementary Figure S6. Plio-Pleistocene evolution of gracile hominin endocranial volume. (a) Pattern of change through time following Miguel and Henneberg (2001). Regression of ln endocranial volume on geological age for 200 specimens yields a long term rate of 0.425 ln units per million years (ca. 1.061×10^{-4} standard deviations per generation) on a time scale of 3.190 m.y. (ca. 127,600 generations). Yellow line traces mean ECV values through geological time. (b) LDI plot of change per unit time for all combinations of differences (standard deviation units) and intervals (generations) in the time series of panel *a*. (c) LRI plot of change per unit time for all combinations of rates (standard deviations per generation) and intervals (generations) in the time series of panel *a*. Note that LDI and LRI slopes of 0.203 and −0.797 differ by one unit: each indicates a time series between expectation for stasis and random change. LDI and LRI intercepts are equal and indicate a step rate of $10^{-0.551} = 0.28$ standard deviations per generation on a time scale of one generation. The long-term difference and rate of change in panel *a* (dashed line) are plotted as open squares in panels *b* and *c*.

Study no. 7. Lee and Wolpoff (2003)

Lee, S.-H. & Wolpoff, M. H. The pattern of evolution in Pleistocene human brain size. *Paleobiology* **29**, 186-196 (2003).

Sang-Hee Lee and Milford Wolpoff compiled information on 94 Pleistocene hominids with endocranial volumes known at the time. All of the specimens analyzed are in the genus *Homo*, including representatives of *Homo erectus*, archaic *Homo sapiens/neandertalensis*, and early modern *Homo sapiens*. These came from 17 successive geological ages. Graphs showing the evolution of cranial capacity were included as Lee and Wolpoff's figures 2 and 3. The first showed geological age in thousands of years plotted on the abscissa, with endocranial volume in cubic centimeters plotted on the ordinate. The second showed geological age in thousands of years on the abscissa and ln ECV in cubic centimeters on the ordinate.

The endocranial volumes and geological ages reported by Lee and Wolpoff (2003) are analyzed here in Figure S7a, where ln ECV (cm^3) is plotted on the abscissa and geological age (m.y.) is plotted on the ordinate. Age is the independent variable and ECV is the dependent variable. Regression of ln ECV on age yields a long-term slope or rate of ln ECV (cm^3) / age (m.y.) = 0.356. Converting to standard deviation units and generations (where the standard deviation of ln ECV = 0.100 cm^3 and one generation = 25 years), this is a long-term rate R = 3.556 std. dev. / 40,000 gen. = 0.000089 std. dev. / gen. — on a time scale or interval I = 1,750,000 / 25 = 70,000 generations. The corresponding difference is $D = R \cdot I = 6.222$ standard deviations. The intercept of ln ECV = 7.21 (cm^3) corresponds to an ECV of 1,353 cm^3.

All of the possible differences between sample mean ECVs in the time series of Figure S7a (expressed in standard deviation units) are plotted against their corresponding intervals (expressed in generations) on the log-difference-interval or LDI graph of Figure S7b. The 136 log differences range in value from −2.484 to 0.923, on log intervals ranging from 3.301 to 4.845. The distribution of differences has a median slope of 0.516, which is significantly different from the slope expected for a stationary time series or purely directional change (asterisks), but not significantly different from the slope expected for a random time series. The median intercept is −1.868, which, exponentiated ($10^{-1.868}$), corresponds to a step difference of 0.014 standard deviations on a time scale of one generation.

All of the possible rates of change between sample mean ECVs in the time series of Figure S7a (in standard deviations per generation) are plotted against their corresponding intervals (generations) on the log-rate-interval or LRI graph of Figure S7c. The 136 log rates range in value from −6.563 to −3.107, on log intervals that again range from 3.301 to 4.845. The distribution of rates has a median slope of 0.484, which is again significantly different from the slope expected for a stationary time series or purely directional change (asterisks), but not significantly different from the slope expected for a random time series. The median intercept is again −1.868, which, exponentiated, corresponds to a step rate h_0 = 0.014 standard deviations per generation on a time scale of one generation.

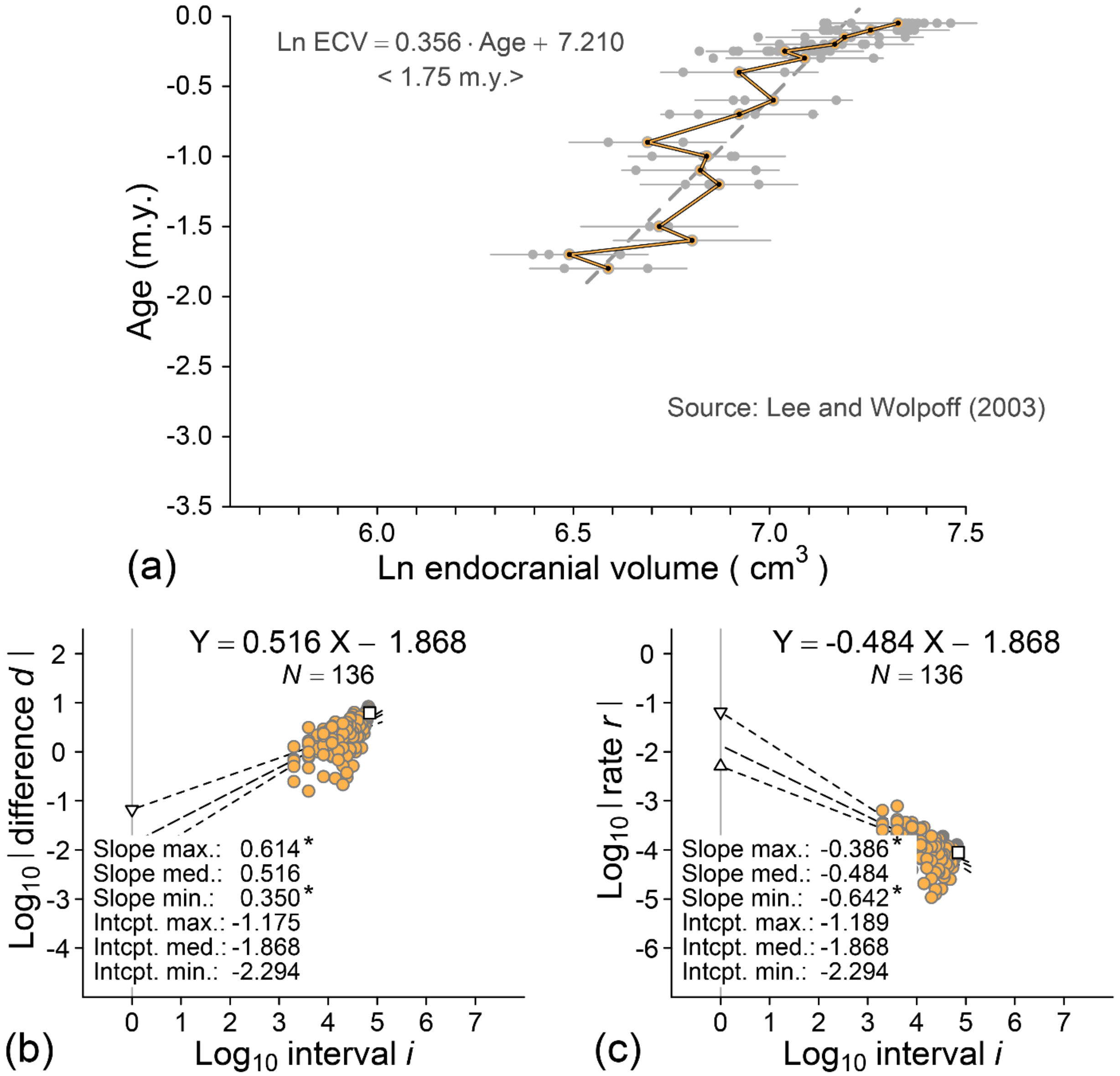

Supplementary Figure S7. Plio-Pleistocene evolution of gracile hominin endocranial volume. (a) Pattern of change through time following Lee and Wolpoff (2003). Regression of ln endocranial volume on geological age for 94 specimens yields a long term rate of 0.356 ln units per million years (ca. 8.889×10^{-5} standard deviations per generation) on a time scale of 1.750 m.y. (ca. 70,000 generations). Yellow line traces mean ECV values through geological time. (b) LDI plot of change per unit time for all combinations of differences (standard deviation units) and intervals (generations) in the time series of panel *a*. (c) LRI plot of change per unit time for all combinations of rates (standard deviations per generation) and intervals (generations) in the time series of panel *a*. Note that LDI and LRI slopes of 0.516 and −0.484 differ by one unit: each indicates a time series of predominantly random change. LDI and LRI intercepts are equal and indicate a step rate of $10^{-1.868} = 0.01$ standard deviations per generation on a time scale of one generation. The long-term difference and rate of change in panel *a* (dashed line) are plotted as open squares in panels *b* and *c*.

Study no. 8. Holloway et al. (2004)

Holloway, R. L., Broadfield, D. C. & Yuan, M. S. *The Human Fossil Record, Volume 3, Brain Endocasts--The Paleoneurological Evidence*. (Wiley-Liss, 2004).

Ralph Holloway and co-authors compiled information on 131 fossil hominids with endocranial volumes known at the time. Gracile forms included *Australopithecus afarensis*, *A. africanus*, *Homo habilis*, *H. ergaster*, *H. rudolfensis*, *H. georgicus*, *H. erectus*, *H. antecessor*, *H. soloensis*, *H. heidelbergensis*, *H. sapiens neandertalensis*, *H. sapiens idaltu*, and *H. sapiens sapiens*, which came from 59 successive geological ages. A graph showing the evolution of cranial capacity was included as Holloway et al.'s figure 140, where geological age in millions of years was plotted on the abscissa and endocranial volume in cubic centimeters was plotted on the ordinate.

The endocranial volumes and geological ages reported by Holloway et al. (2004) are analyzed here in Figure S8a, where ln ECV (cm^3) is plotted on the abscissa and geological age (m.y.) is plotted on the ordinate. Age is the independent variable and ECV is the dependent variable. Regression of ln ECV on age yields a long-term slope or rate of ln ECV (cm^3) / age (m.y.) = 0.391. Converting to standard deviation units and generations (where the standard deviation of ln ECV = 0.100 cm^3 and one generation = 25 years), this is a long-term rate R = 3.908 std. dev. / 40,000 gen. = 0.000098 std. dev. / gen. — on a time scale or interval I = 3,165,000 / 25 = 126,600 generations. The corresponding difference is $D = R \cdot I$ = 12.370 standard deviations. The intercept of ln ECV = 7.252 (cm^3) corresponds to an ECV of 1,411 cm^3.

All of the possible differences between sample mean ECVs in the time series of Figure S8a (expressed in standard deviation units) are plotted against their corresponding intervals (expressed in generations) on the log-difference-interval or LDI graph of Figure S8b. The 1,708 log differences range in value from −3.069 to 1.129, on log intervals ranging from 1.602 to 5.102. The distribution of differences has a median slope of 0.194, which is significantly different from the slopes expected for a stationary time series, random change, or purely directional change (asterisks), but 0.194 is closer to expectation for a stationary time series than it is to expectation for a random walk. The median intercept is −0.548, which, exponentiated ($10^{-0.548}$), corresponds to a step difference of 0.283 standard deviations on a time scale of one generation.

All of the possible rates of change between sample mean ECVs in the time series of Figure S8a (in standard deviations per generation) are plotted against their corresponding intervals (generations) on the log-rate-interval or LRI graph of Figure S8c. The 1,708 log rates range in value from −5.975 to −1.066, on log intervals that again range from 1.602 to 5.102. The distribution of rates has a median slope of −0.806, which is again significantly different from the slopes expected for a stationary time series, random change, or purely directional change (asterisks), but −0.806 is closer to expectation for a stationary time series than it is to expectation for a random walk. The median intercept is again −0.548, which, exponentiated, corresponds to a step rate h_0 = 0.283 standard deviations per generation on a time scale of one generation.

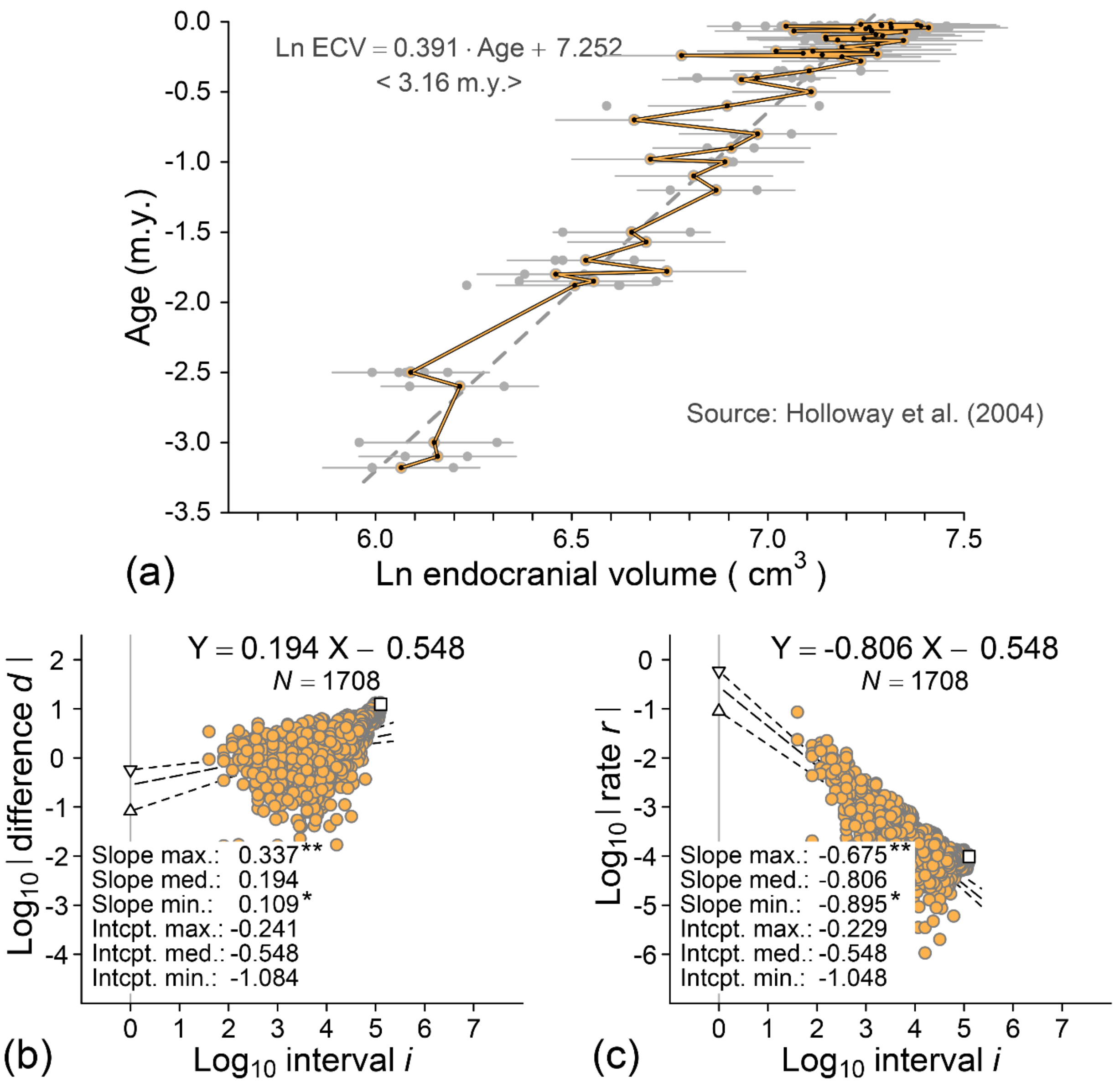

Supplementary Figure S8. Plio-Pleistocene evolution of gracile hominin endocranial volume. (a) Pattern of change through time following Holloway et al. (2004). Regression of ln endocranial volume on geological age for 131 specimens yields a long term rate of 0.390 ln units per million years (ca. 9.745×10^{-5} standard deviations per generation) on a time scale of 3.162 m.y. (ca. 126,480 generations). Yellow line traces mean ECV values through geological time. (b) LDI plot of change per unit time for all combinations of differences (standard deviation units) and intervals (generations) in the time series of panel *a*. (c) LRI plot of change per unit time for all combinations of rates (standard deviations per generation) and intervals (generations) in the time series of panel *a*. Note that LDI and LRI slopes of 0.174 and −0.826 differ by one unit: each indicates a time series between expectation for stasis and random change. LDI and LRI intercepts are equal and indicate a step rate of $10^{-0.466} = 0.34$ standard deviations per generation on a time scale of one generation. The long-term difference and rate of change in panel *a* (dashed line) are plotted as open squares in panels *b* and *c*.

Study no. 9. Ash and Gallup (2007)

Ash, J. & Gallup, G. G. Paleoclimatic variation and brain expansion during human evolution. *Human Nature* **18**, 109-124 (2007).

Jessica Ash and Gordon Gallup compiled information on 109 fossil hominids to test the idea that brain size evolution is related to temperature and distance from the equator. All were in the genus *Homo*, with species including *Homo habilis*, *H. ergaster*, *H. erectus*, *H. antecessor*, *H. heidelbergensis*, archaic *H. sapiens*, and *H. sapiens neandertalensis*. These came from 50 successive geological ages. A graph showing the evolution of cranial capacity was included in Ash and Gallup's figures 1 and 2, where geological age in millions of years was plotted on the abscissa and endocranial volume in cubic centimeters was plotted on the ordinate.

The endocranial volumes and geological ages reported by Ash and Gallup (2007) are analyzed here in Figure S9a, where ln ECV (cm^3) is plotted on the abscissa and geological age (m.y.) is plotted on the ordinate. Age is the independent variable and ECV is the dependent variable. Regression of ln ECV on age yields a long-term slope or rate of ln ECV (cm^3) / age (m.y.) = 0.394. Converting to standard deviation units and generations (where the standard deviation of ln ECV = 0.100 cm^3 and one generation = 25 years), this is a long-term rate R = 3.944 std. dev. / 40,000 gen. = 0.000099 std. dev. / gen. — on a time scale or interval I = 1,865,000 / 25 = 74,600 generations. The corresponding difference is $D = R \cdot I = 7.356$ standard deviations. The intercept of ln ECV = 7.248 (cm^3) corresponds to an ECV of 1,405 cm^3.

All of the possible differences between sample mean ECVs in the time series of Figure S9a (expressed in standard deviation units) are plotted against their corresponding intervals (expressed in generations) on the log-difference-interval or LDI graph of Figure S9b. The 1,225 log differences range in value from −2.373 to 1.072, on log intervals ranging from 1.602 to 4.873. The distribution of differences has a median slope of 0.165, which is significantly different from the slopes expected for a stationary time series, random change, or purely directional change (asterisks), but 0.165 is closer to expectation for a stationary time series than it is to expectation for a random walk. The median intercept is −0.398, which, exponentiated ($10^{-0.398}$), corresponds to a step difference of 0.400 standard deviations on a time scale of one generation.

All of the possible rates of change between sample mean ECVs in the time series of Figure S9a (in standard deviations per generation) are plotted against their corresponding intervals (generations) on the log-rate-interval or LRI graph of Figure S9c. The 1,225 log rates range in value from −6.151 to −1.101, on log intervals that again range from 1.602 to 4.873. The distribution of rates has a median slope of −0.835, which is again significantly different from the slopes expected for a stationary time series, random change, or purely directional change (asterisks), but −0.835 is closer to expectation for a stationary time series than it is to expectation for a random walk. The median intercept is again −0.398, which, exponentiated, corresponds to a step rate h_0 = 0.400 standard deviations per generation on a time scale of one generation.

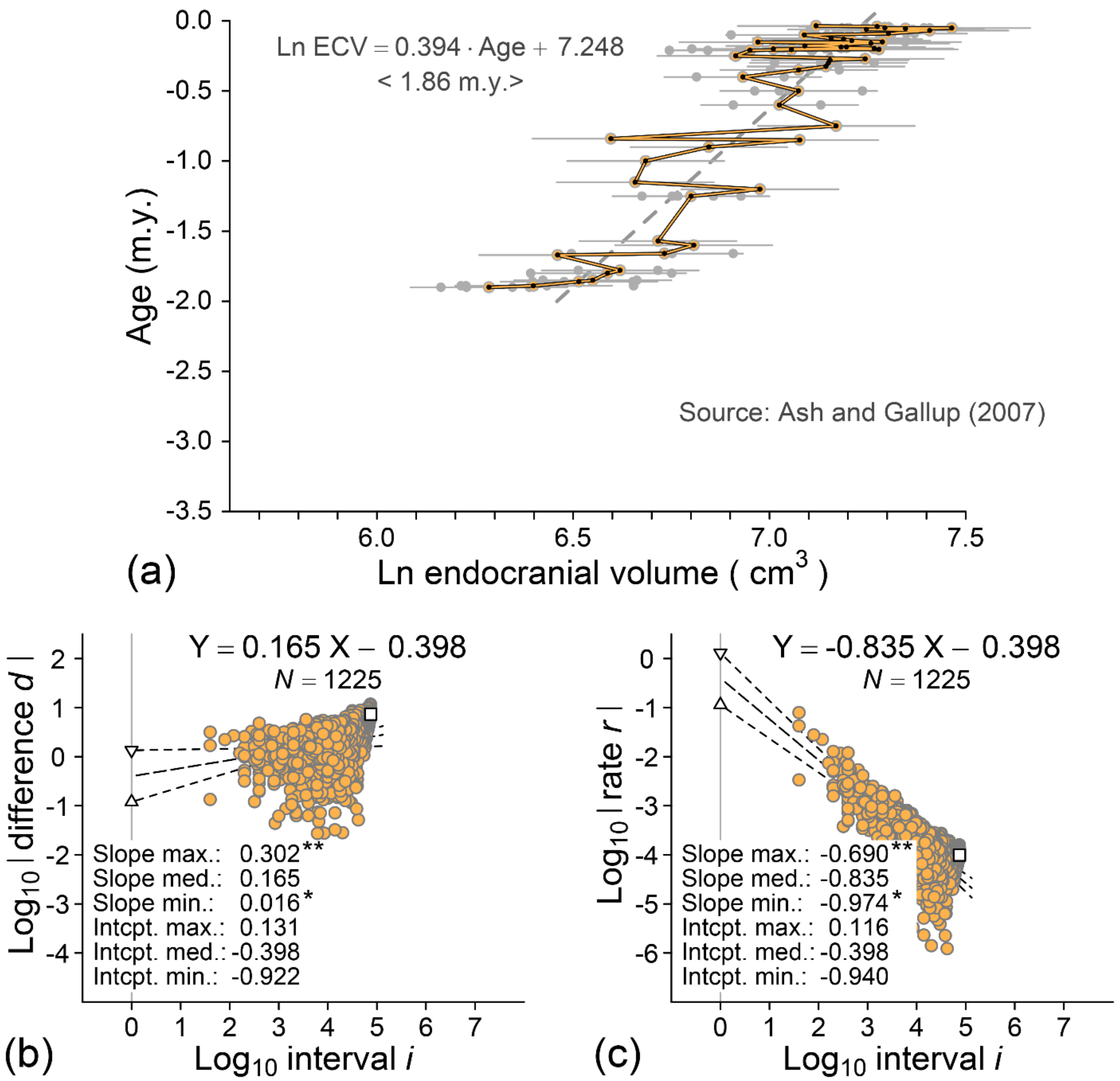

Supplementary Figure S9. Plio-Pleistocene evolution of gracile hominin endocranial volume. (a) Pattern of change through time following Ash and Gallup (2007). Regression of ln endocranial volume on geological age for 109 specimens yields a long term rate of 0.398 ln units per million years (ca. 9.947 $\times 10^{-5}$ standard deviations per generation) on a time scale of 1.860 m.y. (ca. 74,400 generations). Yellow line traces mean ECV values through geological time. (b) LDI plot of change per unit time for all combinations of differences (standard deviation units) and intervals (generations) in the time series of panel *a*. (c) LRI plot of change per unit time for all combinations of rates (standard deviations per generation) and intervals (generations) in the time series of panel *a*. Note that LDI and LRI slopes of 0.184 and −0.816 differ by one unit: each indicates a time series between expectation for stasis and random change. LDI and LRI intercepts are equal and indicate a step rate of $10^{-0.467} = 0.34$ standard deviations per generation on a time scale of one generation. The long-term difference and rate of change in panel *a* (dashed line) are plotted as open squares in panels *b* and *c*.

Study no. 10. Bailey and Geary (2009)

Bailey, D. H. & Geary, D. C. Hominid brain evolution. *Human Nature* **20**, 67-79 (2009).

Drew Bailey and David Geary compiled endocranial volumes and geological ages of 175 fossil hominids, combining information from Ruff et al. (1997), Holloway et al. (2004), and Ash and Gallup (2007). The objective was to compare temperature, equatorial distance, and population density as drivers of brain size increase. The 170 specimens analyzed here, from 66 successive geological ages, all represent the genus *Homo* in one form or another. A graph showing the evolution of cranial capacity was included as Bailey and Geary's figure 1, where geological age in millions of years was plotted on the abscissa and endocranial volume in cubic centimeters was plotted on the ordinate.

The endocranial volumes and geological ages reported by Bailey and Geary (2009) are analyzed here in Figure S10a, where ln ECV (cm^3) is plotted on the abscissa and geological age (m.y.) is plotted on the ordinate. Age is the independent variable and ECV is the dependent variable. Regression of ln ECV on age yields a long-term slope or rate of ln ECV (cm^3) / age (m.y.) = 0.402. Converting to standard deviation units and generations (where the standard deviation of ln ECV = 0.100 cm^3 and one generation = 25 years), this is a long-term rate R = 4.022 std. dev. / 40,000 gen. = 0.000100 std. dev. / gen. — on a time scale or interval I = 1,880,000 / 25 = 75,200 generations. The corresponding difference is $D = R \cdot I$ = 7.562 standard deviations. The intercept of ln ECV = 7.274 (cm^3) corresponds to an ECV of 1,442 cm^3.

All of the possible differences between sample mean ECVs in the time series of Figure S10a (expressed in standard deviation units) are plotted against their corresponding intervals (expressed in generations) on the log-difference-interval or LDI graph of Figure S10b. The 2,145 log differences range in value from −2.605 to 1.005, on log intervals ranging from 1.602 to 4.876. The distribution of differences has a median slope of 0.228, which is significantly different from the slopes expected for a stationary time series, random change, or purely directional change (asterisks), but 0.228 is closer to expectation for a stationary time series than it is to expectation for a random walk. The median intercept is −0.684, which, exponentiated ($10^{-0.684}$), corresponds to a step difference of 0.207 standard deviations on a time scale of one generation.

All of the possible rates of change between sample mean ECVs in the time series of Figure S10a (in standard deviations per generation) are plotted against their corresponding intervals (generations) on the log-rate-interval or LRI graph of Figure S10c. The 2,145 log rates range in value from −6.947 to −1.101, on log intervals that again range from 1.602 to 4.876. The distribution of rates has a median slope of −0.772, which is again significantly different from the slopes expected for a stationary time series, random change, or purely directional change (asterisks), but −0.772 is closer to expectation for a stationary time series than it is to expectation for a random walk. The median intercept is again −0.684, which, exponentiated, corresponds to a step rate h_0 = 0.207 standard deviations per generation on a time scale of one generation.

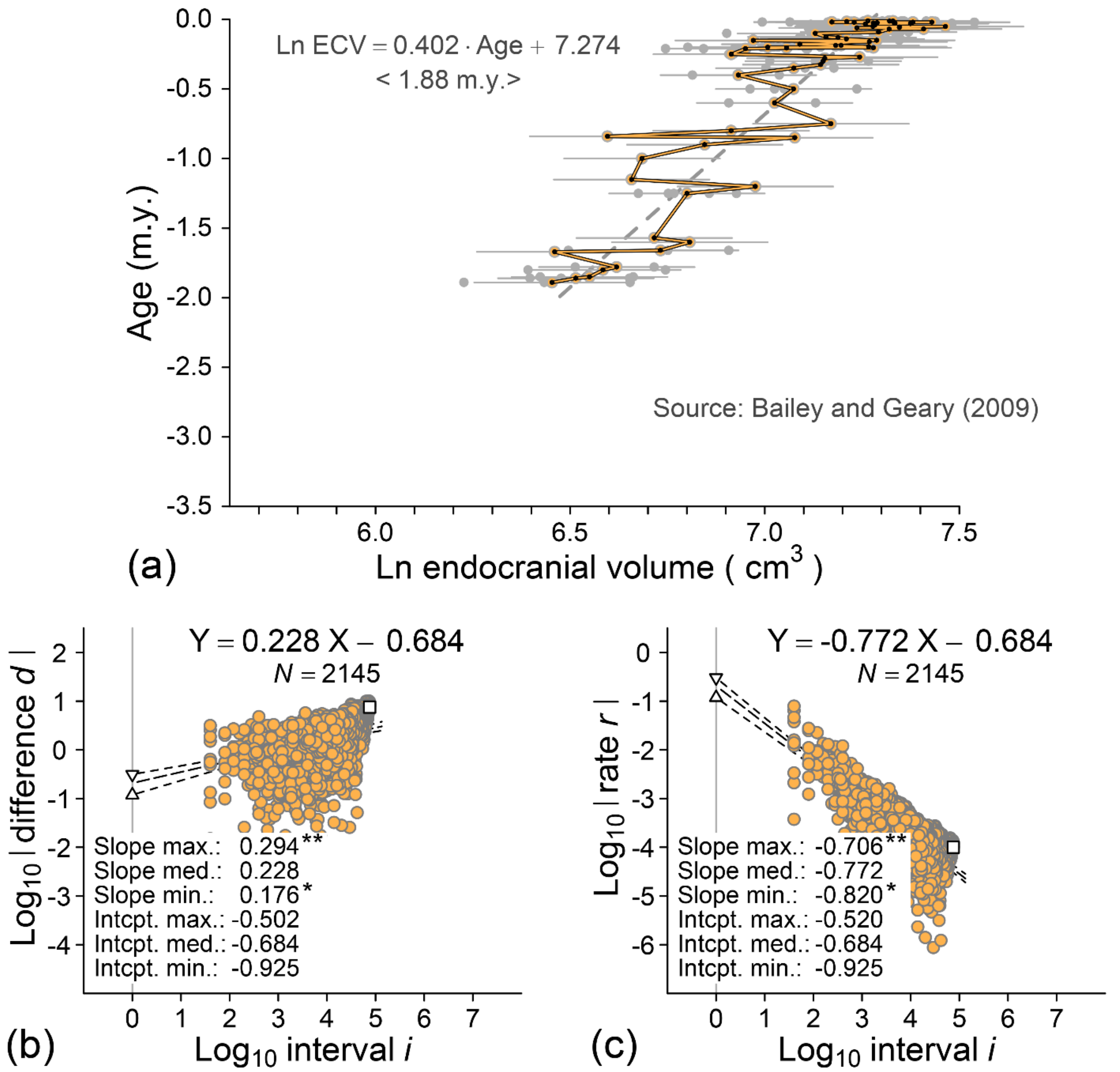

Supplementary Figure S10. Plio-Pleistocene evolution of gracile hominin endocranial volume. (a) Pattern of change through time following Bailey and Geary (2009). Regression of ln endocranial volume on geological age for 170 specimens yields a long term rate of 0.402 ln units per million years (ca. 1.006 $\times 10^{-4}$ standard deviations per generation) on a time scale of 1.879 m.y. (ca. 75,160 generations). Yellow line traces mean ECV values through geological time. (b) LDI plot of change per unit time for all combinations of differences (standard deviation units) and intervals (generations) in the time series of panel *a*. (c) LRI plot of change per unit time for all combinations of rates (standard deviations per generation) and intervals (generations) in the time series of panel *a*. Note that LDI and LRI slopes of 0.233 and −0.767 differ by one unit: each indicates a time series between expectation for stasis and random change. LDI and LRI intercepts are equal and indicate a step rate of $10^{-0.697} = 0.20$ standard deviations per generation on a time scale of one generation. The long-term difference and rate of change in panel *a* (dashed line) are plotted as open squares in panels *b* and *c*.

Study no. 11. Shultz et al. (2012)

Shultz, S., Nelson, E. & Dunbar, R. I. M. Hominin cognitive evolution: identifying patterns and processes in the fossil and archaeological record. *Philosophical Transactions of the Royal Society B: Biological Sciences* **367**, 2130-2140 (2012).

Susanne Shultz and co-authors compiled information on fossil hominids with endocranial volumes known at the time. Their principal source was the dataset of Bailey and Geary (2009), with numerous additions and subtractions explained in their supplementary information. The objective was further evaluation of paleoclimate as a driver of human brain-size evolution. A total of 174 specimens are included in the analysis here, representing *Australopithecus afarensis, A. africanus*, *Homo habilis*, *H. rudolfensis*, *H. ergaster*, *H. georgicus*, *Homo erectus*, *H. heidelbergensis*, *H. neandertalensis*, and early *H. sapiens*. These came from 88 successive geological ages. A graph showing the evolution of cranial capacity was included as Shultz et al.'s figure 1, where geological age in millions of years was plotted on the abscissa, binned in 100 k.y. bins, and $\log_{10}$ endocranial volume in cubic centimeters was plotted on the ordinate.

The endocranial volumes and geological ages reported by Shultz et al. (2012) are analyzed in Figure S11a, where ln ECV (cm^3) is plotted on the abscissa and geological age (m.y.) is plotted on the ordinate. Age is the independent variable and ECV is the dependent variable. Regression of ln ECV on age yields a long-term slope or rate of ln ECV (cm^3) / age (m.y.) = 0.415. Converting to standard deviation units and generations (where the standard deviation of ln ECV = 0.100 cm^3 and one generation = 25 years), this is a long-term rate R = 4.153 std. dev. / 40,000 gen. = 0.000104 std. dev. / gen. — on a time scale or interval I = 3,190,000 / 25 = 127,600 generations. The corresponding difference is $D = R \cdot I$ = 13.249 standard deviations. The intercept of ln ECV = 7.284 (cm^3) corresponds to an ECV of 1,457 cm^3.

All of the possible differences between sample mean ECVs in the time series of Figure S11a (expressed in standard deviation units) are plotted against their corresponding intervals (expressed in generations) on the log-difference-interval or LDI graph of Figure S11b. The 3,825 log differences range in value from −3.221 to 1.190, on log intervals ranging from 1.602 to 5.106. The distribution of differences has a median slope of 0.290, which is significantly different from the slopes expected for a stationary time series, random change, or purely directional change (asterisks). A slope of 0.290 is closer to expectation for a random-walk time series (0.500) than it is to either endpoint (1.000 or 0.000). The median intercept is −0.878, which, exponentiated ($10^{-0.878}$), corresponds to a step difference of 0.132 standard deviations on a time scale of one generation.

All of the possible rates of change between sample mean ECVs in the time series of Figure S11a (in standard deviations per generation) are plotted against their corresponding intervals (generations) on the log-rate-interval or LRI graph of Figure S11c. The 3,825 log rates range in value from −7.011 to −1.209, on log intervals that again range from 1.602 to 5.106. The distribution of rates has a median slope of −0.710, which is again significantly different from the slopes expected for a stationary time series, random change, or purely directional change (asterisks). A slope of −0.710 is closer to expectation for a random-walk time series (−0.500) than it is to either endpoint (0.000 or −1.000). The median intercept is again −0.878, which, exponentiated, corresponds to a step rate h_0 = 0.132 standard deviations per generation on a time scale of one generation.

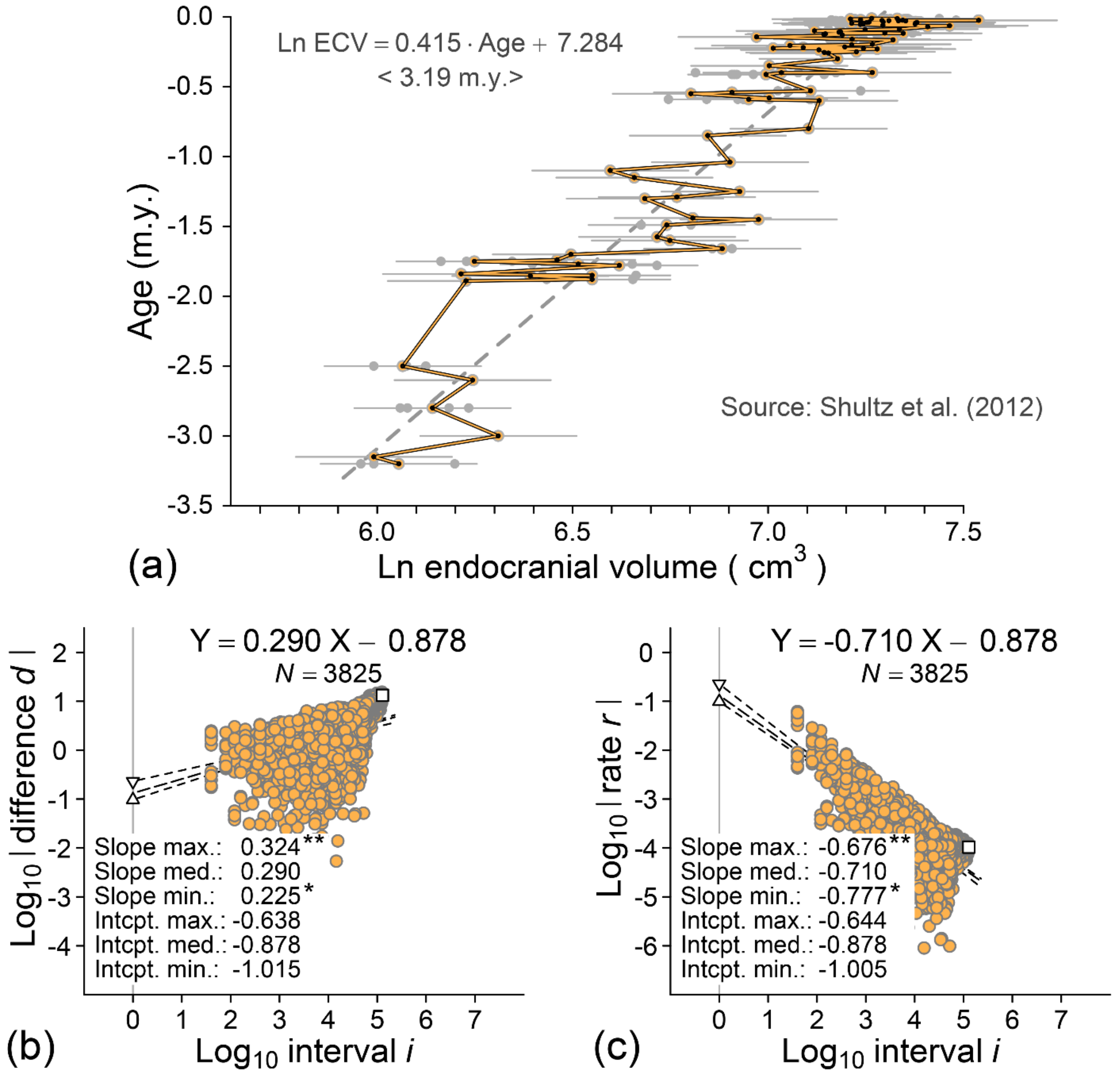

Supplementary Figure S11. Plio-Pleistocene evolution of gracile hominin endocranial volume. (a) Pattern of change through time following Shultz et al. (2012). Regression of ln endocranial volume on geological age for 174 specimens yields a long term rate of 0.415 ln units per million years (ca. 1.039×10^{-4} standard deviations per generation) on a time scale of 3.188 m.y. (ca. 127,520 generations). Yellow line traces mean ECV values through geological time. (b) LDI plot of change per unit time for all combinations of differences (standard deviation units) and intervals (generations) in the time series of panel *a*. (c) LRI plot of change per unit time for all combinations of rates (standard deviations per generation) and intervals (generations) in the time series of panel *a*. Note that LDI and LRI slopes of 0.290 and −0.710 differ by one unit: each indicates a time series between expectation for stasis and random change. LDI and LRI intercepts are equal and indicate a step rate of $10^{-0.872} = 0.13$ standard deviations per generation on a time scale of one generation. The long-term difference and rate of change in panel *a* (dashed line) are plotted as open squares in panels *b* and *c*.

Study no. 12. Schoenemann (2013)

Schoenemann, P. T. in *A Companion to Paleoanthropology* (ed Begun, D. R.) 136-164 (Blackwell, 2013).

Thomas Schoenemann compiled information on 166 mature fossil hominids from 74 successive intervals of geological time, including representatives of the species *Australopithecus afarensis*, *A. africanus*, *Homo habilis*, *H. ergaster*, *H. rudolfensis*, *H. georgicus*, *H. erectus*, *H. antecessor*, *H. soloensis*, *H. heidelbergensis*, *H. sapiens neandertalensis*, and *H. sapiens sapiens*. Endocranial volumes and geological ages for these taxa were taken from Holloway et al. (2004) and other souces. Schoenemann included a graph showing the evolution of cranial capacity as figure 8.1b, where $\log_{10}$ of geological age in millions of years was plotted on the abscissa and endocranial volume in cubic centimeters was plotted on the ordinate.

The endocranial volumes and geological ages for gracile hominins reported by Schoenemann (2013) are analyzed here in Figure S12a, where ln ECV (cm^3) is plotted on the abscissa and geological age (m.y.) is plotted on the ordinate. Age is the independent variable and ECV is the dependent variable. Regression of ln ECV on age yields a long-term slope or rate of ln ECV (cm^3) / age (m.y.) = 0.382. Converting to standard deviation units and generations (where the standard deviation of ln ECV = 0.100 cm^3 and one generation = 25 years), this is a long-term rate R = 3.816 std. dev. / 40,000 gen. = 0.000095 std. dev. / gen. — on a time scale or interval I = 3,170,000 / 25 = 126,800 generations. The corresponding difference is $D = R \cdot I$ = 12.095 standard deviations. The intercept of ln ECV = 7.248 (cm^3) corresponds to an ECV of 1,405 cm^3.

All of the possible differences between sample mean ECVs in the time series of Figure S12a (expressed in standard deviation units) are plotted against their corresponding intervals (expressed in generations) on the log-difference-interval or LDI graph of Figure S12b. The 2,695 log differences range in value from −2.469 to 1.121, on log intervals ranging from 1.602 to 5.103. The distribution of differences has a median slope of 0.256, which is significantly different from the slopes expected for a stationary time series, random change, or purely directional change (asterisks). A slope of 0.256 is closer to expectation for a random-walk time series (0.500) than it is to either endpoint (1.000 or 0.000). The median intercept is −0.761, which, exponentiated ($10^{-0.761}$), corresponds to a step difference of 0.173 standard deviations on a time scale of one generation.

All of the possible rates of change between sample mean ECVs in the time series of Figure S12a (in standard deviations per generation) are plotted against their corresponding intervals (generations) on the log-rate-interval or LRI graph of Figure S12c. The 2,695 log rates range in value from −6.204 to −1.461, on log intervals that again range from 1.602 to 5.103. The distribution of rates has a median slope of −0.744, which is again significantly different from the slopes expected for a stationary time series, random change, or purely directional change (asterisks). A slope of −0.744 is closer to expectation for a random-walk time series (−0.500) than it is to either endpoint (0.000 or −1.000). The median intercept is again −0.761, which, exponentiated, corresponds to a step rate h_0 = 0.173 standard deviations per generation on a time scale of one generation.

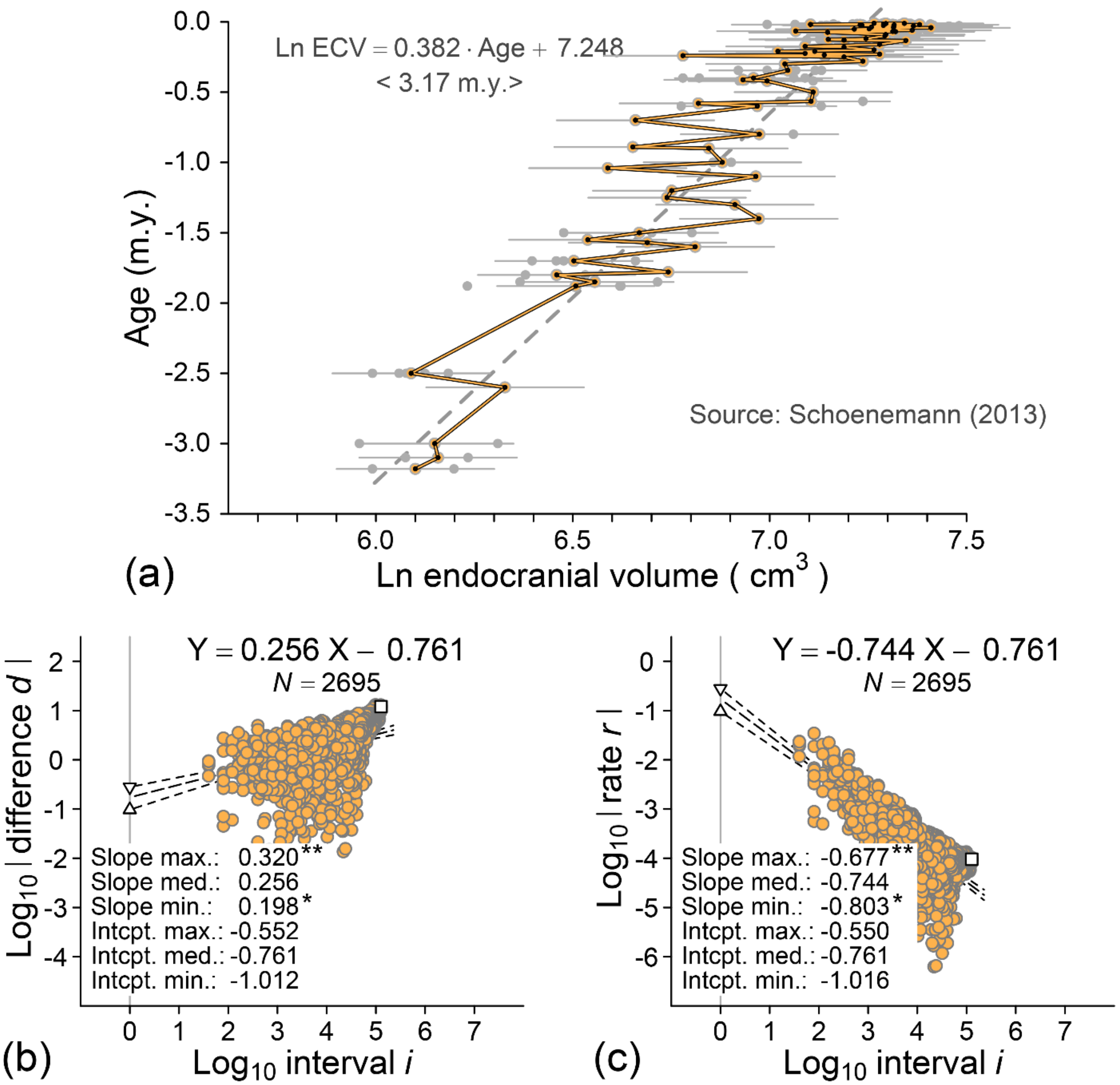

Supplementary Figure S12. Plio-Pleistocene evolution of gracile hominin endocranial volume. (a) Pattern of change through time following Schoenemann (2013). Regression of ln endocranial volume on geological age for 166 specimens yields a long term rate of 0.381 ln units per million years (ca. 9.532×10^{-5} standard deviations per generation) on a time scale of 3.169 m.y. (ca. 126,760 generations). Yellow line traces mean ECV values through geological time. (b) LDI plot of change per unit time for all combinations of differences (standard deviation units) and intervals (generations) in the time series of panel *a*. (c) LRI plot of change per unit time for all combinations of rates (standard deviations per generation) and intervals (generations) in the time series of panel *a*. Note that LDI and LRI slopes of 0.255 and −0.745 differ by one unit: each indicates a time series between expectation for stasis and random change. LDI and LRI intercepts are equal and indicate a step rate of $10^{-0.755} = 0.18$ standard deviations per generation on a time scale of one generation. The long-term difference and rate of change in panel *a* (dashed line) are plotted as open squares in panels *b* and *c*.

Study no. 13. Du et al. (2018)

Du, A. *et al.* Pattern and process in hominin brain size evolution are scale-dependent. *Proceedings of the Royal Society B: Biological Sciences* **285**, 20172738 (2018).

Andrew Du and co-authors compiled information on 75 fossil hominins and endocranial volumes coming from 46 successive geological ages. Gracile forms included *Australopithecus afarensis*, *A. africanus*, *Homo rudolfensis*, *H. habilis*, *H. ergaster*. *H. georgicus*, *H. erectus*, and *H. heidelbergensis*. A graph showing the evolution of cranial capacity was included as Du et al.'s figure 2, where geological age in millions of years was plotted on the abscissa and $\log_{10}$ endocranial volume in cubic centimeters was plotted on the ordinate.

The endocranial volumes and geological ages reported by Du et al. (2018) are analyzed here in Figure S13a, where ln ECV (cm^3) is plotted on the abscissa and geological age (m.y.) is plotted on the ordinate. Age is the independent variable and ECV is the dependent variable. Regression of ln ECV on age yields a long-term slope or rate of ln ECV (cm^3) / age (m.y.) = 0.374. Converting to standard deviation units and generations (where the standard deviation of ln ECV = 0.100 cm^3 and one generation = 25 years), this is a long-term rate R = 3.743 std. dev. / 40,000 gen. = 0.000094 std. dev. / gen. — on a time scale or interval I = 2,875,500 / 25 = 115,020 generations. The corresponding difference is $D = R \cdot I$ = 10.762 standard deviations. The intercept of ln ECV = 7.192 (cm^3) corresponds to an ECV of 1,329 cm^3.

All of the possible differences between sample mean ECVs in the time series of Figure S13a (expressed in standard deviation units) are plotted against their corresponding intervals (expressed in generations) on the log-difference-interval or LDI graph of Figure S13b. The 1,034 log differences range in value from −2.141 to 1.085, on log intervals ranging from 2.000 to 5.061. The distribution of differences has a median slope of 0.202, which is significantly different from the slopes expected for a stationary time series, random change, or purely directional change (asterisks), but 0.202 is closer to expectation for a stationary time series than it is to expectation for a random walk. The median intercept is −0.514, which, exponentiated ($10^{-0.514}$), corresponds to a step difference of 0.306 standard deviations on a time scale of one generation.

All of the possible rates of change between sample mean ECVs in the time series of Figure S13a (in standard deviations per generation) are plotted against their corresponding intervals (generations) on the log-rate-interval or LRI graph of Figure S13c. The 1,034 log rates range in value from −6.522 to −1.908, on log intervals that again range from 2.000 to 5.061. The distribution of rates has a median slope of −0.798, which is again significantly different from the slopes expected for a stationary time series, random change, or purely directional change (asterisks), but −0.798 is closer to expectation for a stationary time series than it is to expectation for a random walk. The median intercept is again −0.514, which, exponentiated, corresponds to a step rate h_0 = 0.306 standard deviations per generation on a time scale of one generation.

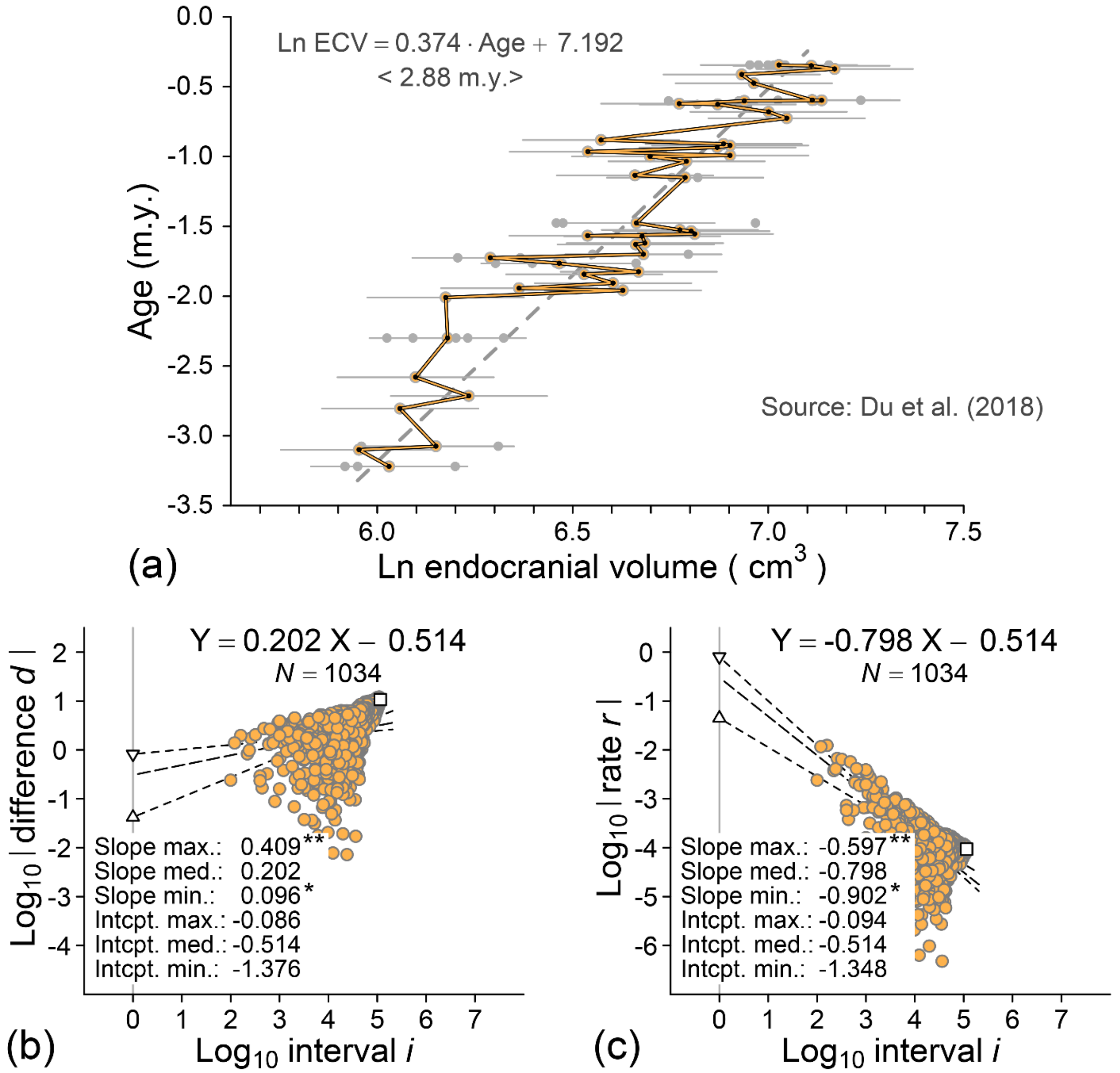

Supplementary Figure S13. Plio-Pleistocene evolution of gracile hominin endocranial volume. (a) Pattern of change through time following Du et al. (2018). Regression of ln endocranial volume on geological age for 75 specimens yields a long term rate of 0.374 ln units per million years (ca. 9.356×10^{-5} standard deviations per generation) on a time scale of 2.876 m.y. (ca. 115,020 generations). Yellow line traces mean ECV values through geological time. (b) LDI plot of change per unit time for all combinations of differences (standard deviation units) and intervals (generations) in the time series of panel *a*. (c) LRI plot of change per unit time for all combinations of rates (standard deviations per generation) and intervals (generations) in the time series of panel *a*. Note that LDI and LRI slopes of 0.202 and −0.798 differ by one unit: each indicates a time series between expectation for stasis and random change. LDI and LRI intercepts are equal and indicate a step rate of $10^{-0.514} = 0.31$ standard deviations per generation on a time scale of one generation. The long-term difference and rate of change in panel *a* (dashed line) are plotted as open squares in panels *b* and *c*.

Study no. 14. Ponce de León et al. (2021)

Ponce de León, M. S. *et al.* The primitive brain of early *Homo*. *Science* **372**, 165-171 (2021).

Marcia Ponce de León and co-authors provided information for 38 fossil specimens with endocranial volumes identified as early *Homo*. Middle and late Pleistocene *Homo naledi* and *Homo floresiensis*, representing distinctively small lineages, are omitted from analysis here. The rest came from 18 successive geological ages. A graph showing the evolution of cranial capacity was included as Ponce de León et al.'s figure 4a, where geological age in millions of years is plotted on the abscissa and endocranial volume in cubic centimeters is plotted on the ordinate.

The endocranial volumes and geological ages reported by Ponce de León et al. (2021) are analyzed here in Figure S14a, where ln ECV (cm^3) is plotted on the abscissa and geological age (m.y.) is plotted on the ordinate. Age is the independent variable and ECV is the dependent variable. Regression of ln ECV on age yields a long-term slope or rate of ln ECV (cm^3) / age (m.y.) = 0.339. Converting to standard deviation units and generations (where the standard deviation of ln ECV = 0.100 cm^3, and one generation = 25 years), this is a long-term rate R = 3.393 std. dev. / 40,000 gen. = 0.000085 std. dev. / gen. — on a time scale or interval I = 1,917,500 / 25 = 76,700 generations. The corresponding difference is $D = R \cdot I = 6.507$ standard deviations. The intercept of ln ECV = 7.154 (cm^3) corresponds to an ECV of 1,279 cm^3.

All of the possible differences between sample mean ECVs in the time series of Figure S14a (expressed in standard deviation units) are plotted against their corresponding intervals (expressed in generations) on the log-difference-interval or LDI graph of Figure S14b. The 153 log differences range in value from −1.455 to 0.981, on log intervals ranging from 2.602 to 4.885. The distribution of differences has a median slope of 0.101, which is significantly different from the slopes expected for random or directional change (asterisks), but not significantly different from the slope of 0.000 expected for a stationary time series. The median intercept is −0.090, which, exponentiated ($10^{-0.090}$), corresponds to a step difference of 0.814 standard deviations on a time scale of one generation.

All of the possible rates of change between sample mean ECVs in the time series of Figure S14a (in standard deviations per generation) are plotted against their corresponding intervals (generations) on the log-rate-interval or LRI graph of Figure S14c. The 153 log rates range in value from −5.756 to −2.262, on log intervals that again range from 2.602 to 4.885. The distribution of rates has a median slope of −0.899, which is again significantly different from the slopes expected for random or directional change (asterisks), but not significantly different from the slope of −1.000 expected for a stationary time series. The median intercept is again −0.090, which, exponentiated, corresponds to a step rate h_0 = 0.814 standard deviations per generation on a time scale of one generation.

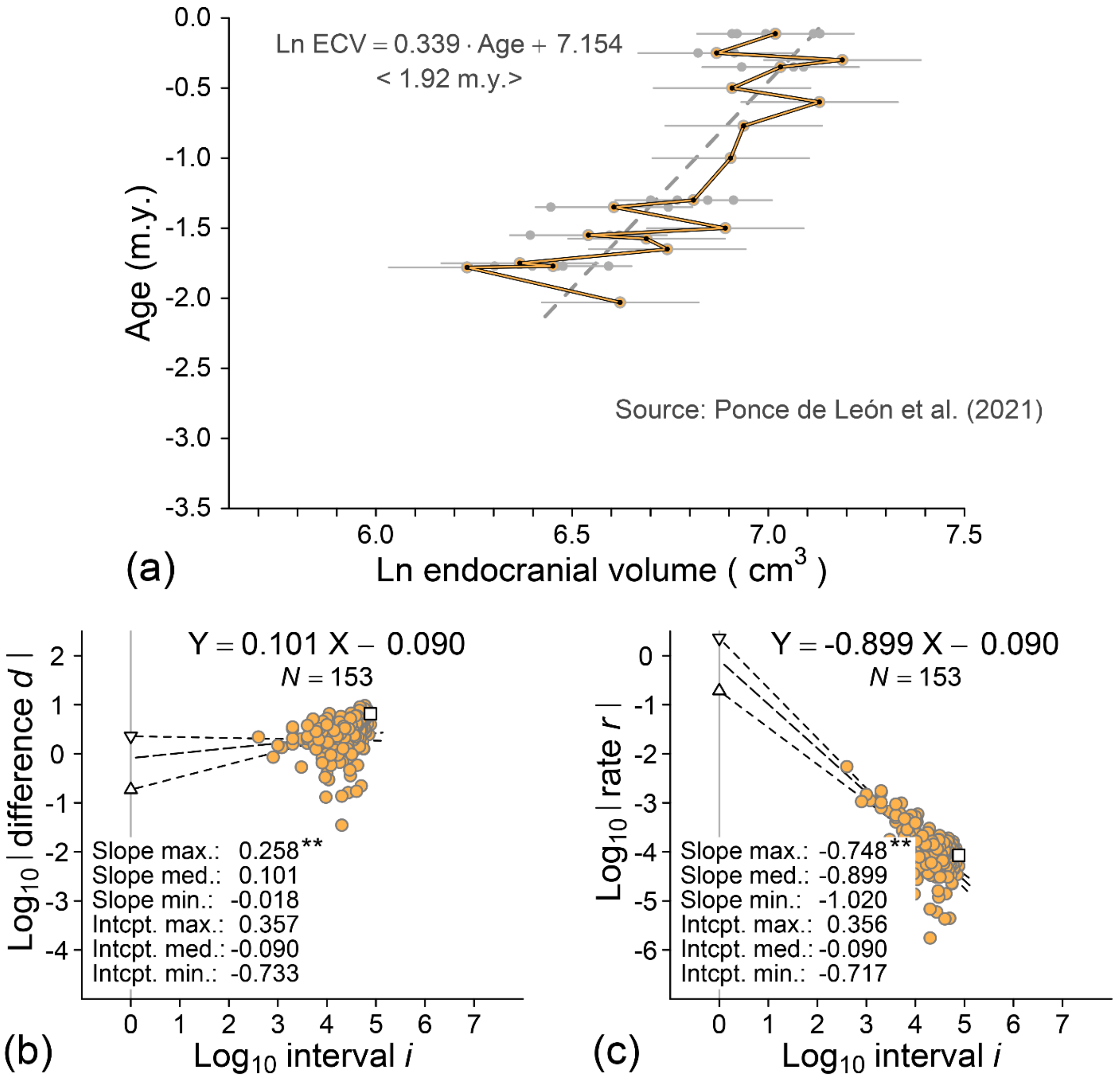

Supplementary Figure S14. Pleistocene evolution of early *Homo* endocranial volume. (a) Pattern of change through time following Ponce de León et al. (2021). Regression of ln endocranial volume on geological age for 36 specimens yields a long term rate of 0.339 ln units per million years (ca. 8.484×10^{-5} standard deviations per generation) on a time scale of 1.918 m.y. (ca. 76,700 generations). Yellow line traces mean ECV values through geological time. (b) LDI plot of change per unit time for all combinations of differences (standard deviation units) and intervals (generations) in the time series of panel *a*. (c) LRI plot of change per unit time for all combinations of rates (standard deviations per generation) and intervals (generations) in the time series of panel *a*. Note that LDI and LRI slopes of 0.101 and −0.899 differ by one unit: each indicates a time series significantly different from directional and random change (asterisks), but insignificantly different from stasis. LDI and LRI intercepts are equal and indicate a step rate of $10^{-0.090} = 0.814$ standard deviations per generation on a time scale of one generation. The long-term difference and rate of change in panel *a* (dashed line) are plotted as open squares in panels *b* and *c*.

Additional Supplementary Figures

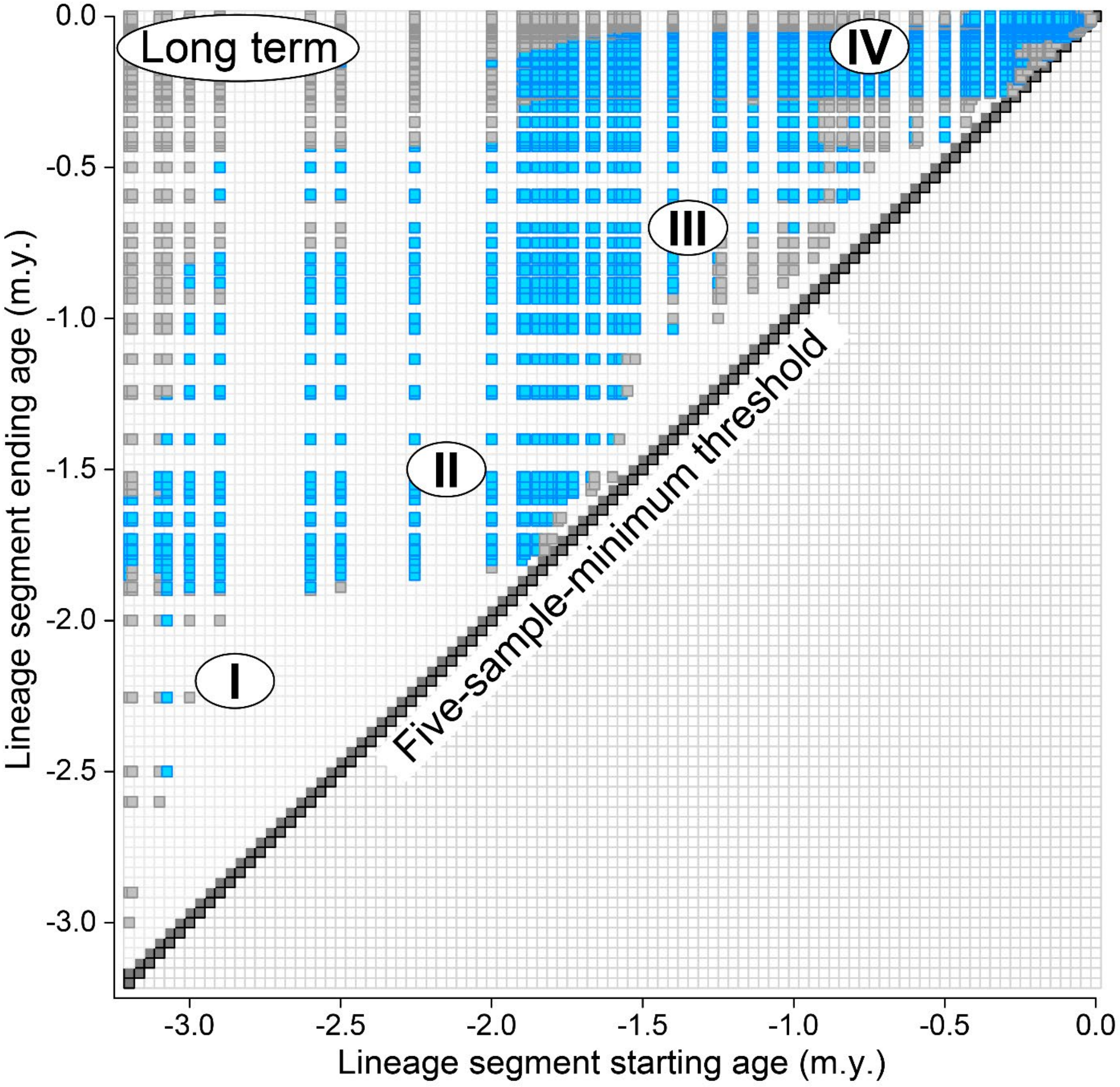

Supplementary Figure S15. Evaluation of evolutionary mode for all 98 ages and corresponding time series segments in the consensus *Australopithecus–Homo* lineage of Figure 1 in the main text. The starting age for a segment is shown on the abscissa and the ending age is shown on the ordinate (all lie above the five-sample-minimum diagonal). Empty bins represent missing segments. Each time series segment was analyzed following the protocol illustrated in Figure 1c of the main text. Gray squares are time series segments with LRI temporal scaling slopes that cannot be distinguished from −0.500 expected for random change. Blue squares are time series segments that can be distinguished statistically from random and directional change but not from stasis. No directional segments are evident at this scale. Time series phases I through IV are those identified in Figure 3 of the main text, with II and IV being more distinct from random than I and III. Long-term change here, averaging change on shorter time scales, cannot be distinguished from random.

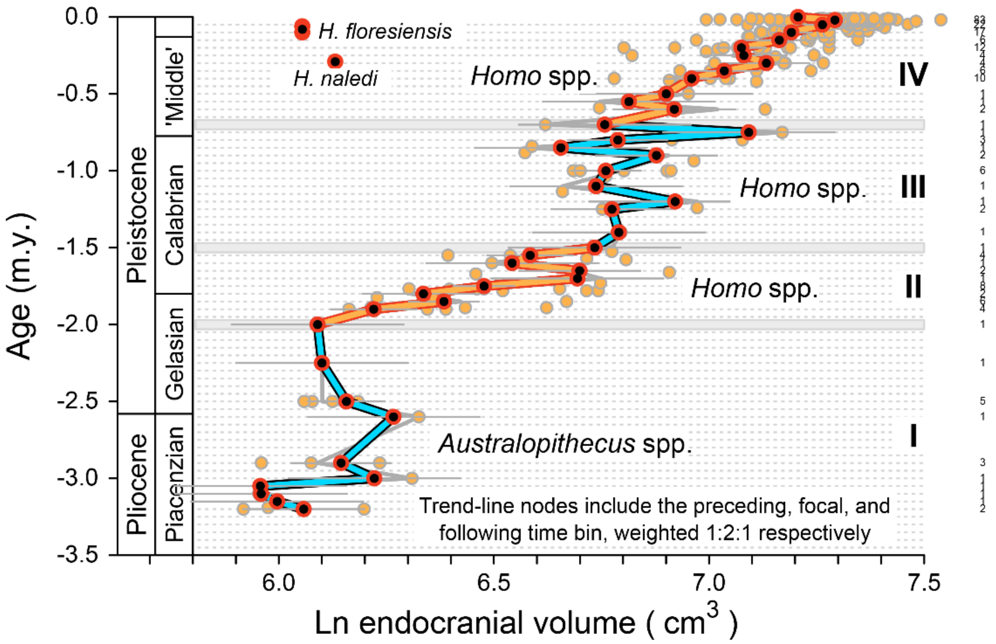

Supplementary Figure S16. Consensus pattern of brain size evolution in Plio-Pleistocene hominins binned in 0.05 million year (50 k.y.) time bins. Points shown in the background are median ln ECV values and ages for 233 specimens listed in Supplementary Dataset 1. Underlying gray line traces mean ln ECV values through time for 39 successive bins. Blue and yellow lines trace a 1:2:1 weighted running mean ln ECV value through sequential time bins. Sample sizes (consensus number of specimens) for each bin are listed in the right-hand column. As in Figure 3 of the main text, four phases of change in ln ECV values are evident: I, in blue, an initial phase of relatively stationary brain size; II, in yellow, an early phase of increasing brain size; III, in blue, a second phase of relatively stationary brain size; and IV, in yellow, a second phase of increasing brain size. Differences between successive segments of the principal *Australopithecus–Homo* time series are evaluated statistically in the following Figure S17.

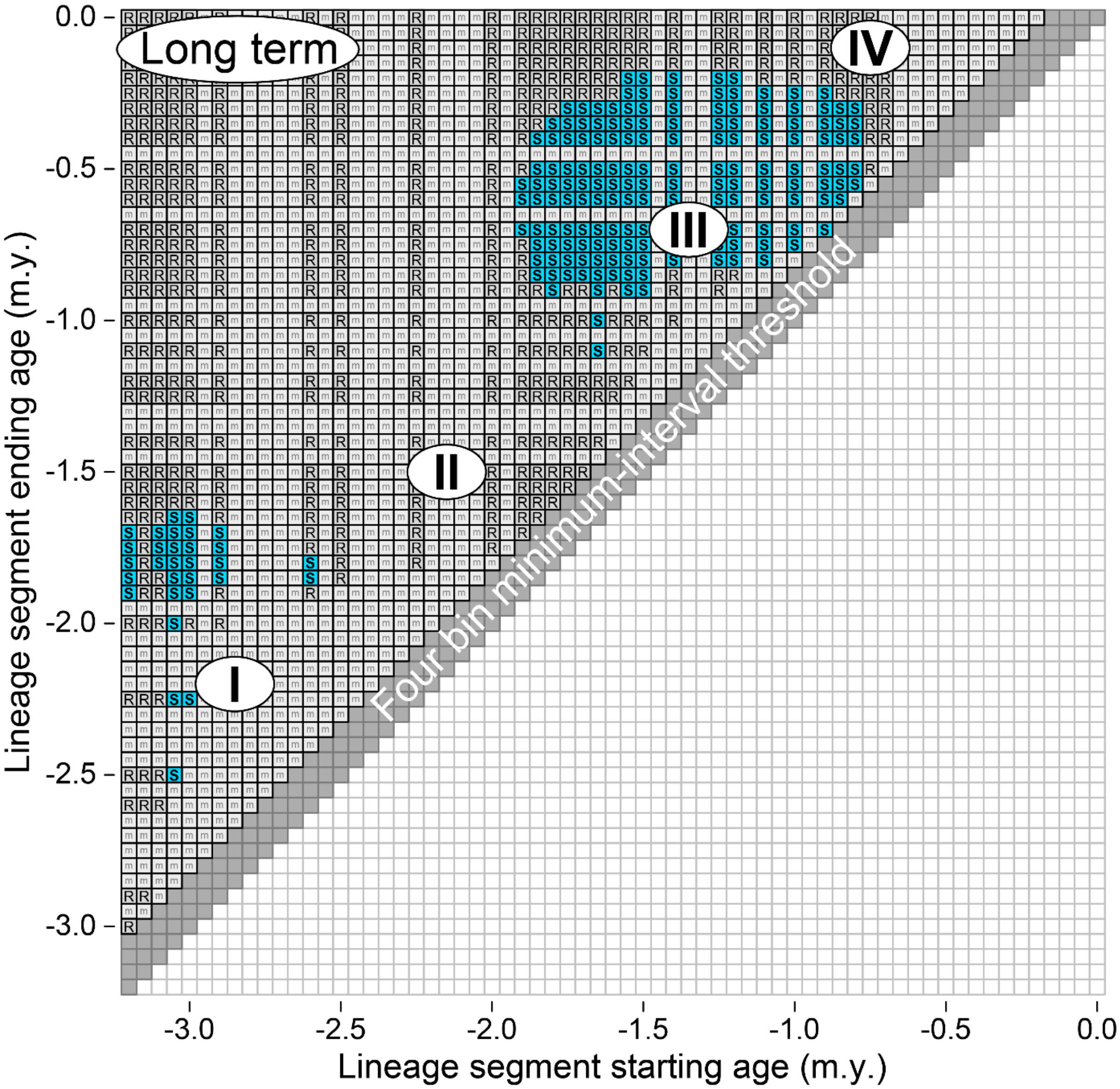

Supplementary Figure S17. Evaluation of evolutionary mode for all time series segments in the binned and smoothed *Australopithecus–Homo* lineage shown above in Supplementary Figure S16. Specimens were binned by age into successive 0.05 m.y. (50 k.y.) bins. Starting age for a segment is shown on the abscissa and ending age is shown on the ordinate. Empty bin segments are marked 'm' for missing. Each segment was analyzed following the protocol illustrated in Figure 1b in the main text. Time series segments with LRI temporal scaling slopes that cannot be distinguished from −0.500 expected for random change are marked 'R'. Time series segments that can be distinguished statistically from random and directional change but not from stasis are shown in blue ('S'). When aggregated in 50 k.y. bins no directional segments can be distinguished from stasis or random change. Minimum segment length required for statistical significance is five 50-k.y. time bins. Note that time series phases I through IV identified in the main text emerge as distinct modes, but only I and III can be distinguished from random. Long-term change is an average of change on shorter time scales.

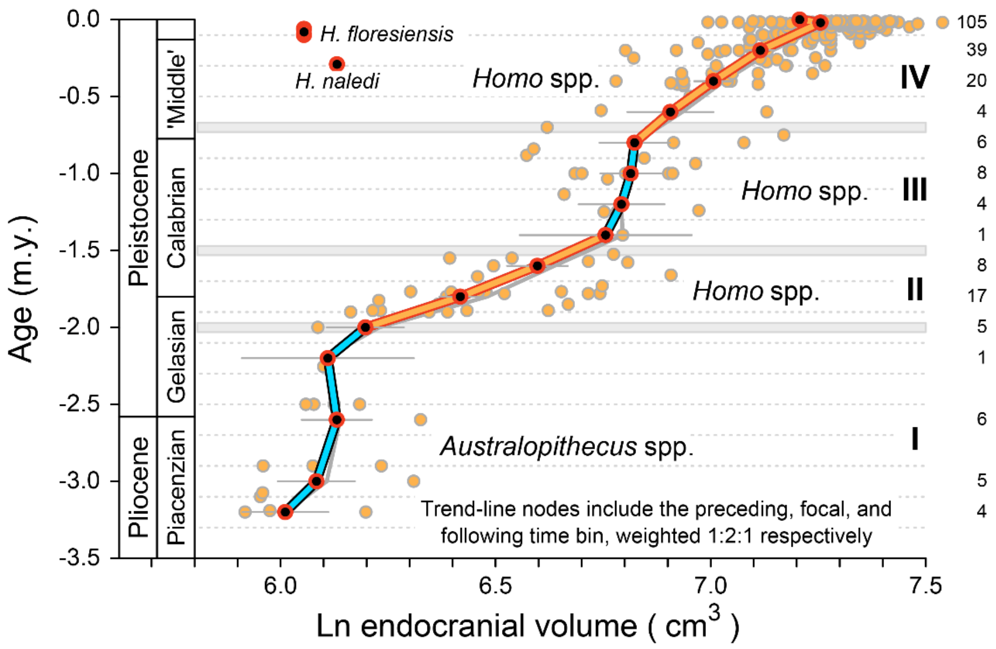

Supplementary Figure S18. Consensus pattern of brain size evolution in Plio-Pleistocene hominins binned in 0.2 million year (200 k.y.) time bins. Points shown in the background are median ln ECV values and ages for 233 specimens listed in Supplementary Dataset 1. Underlying gray line traces mean ln ECV values through time for 15 successive time bins. Blue and yellow lines trace a 1:2:1 weighted running mean ln ECV value through sequential time bins. Sample sizes (consensus number of specimens) for each bin are listed in the right-hand column. As in Figure 3 of the main text, four phases of change in ln ECV values are evident: I, in blue, an initial phase of relatively stationary brain size; II, in yellow, an early phase of increasing brain size; III, in blue, a second phase of relatively stationary brain size; and IV, in yellow, a second phase of increasing brain size. Differences between successive segments of the principal *Australopithecus–Homo* time series are evaluated statistically in the following Figure S19.

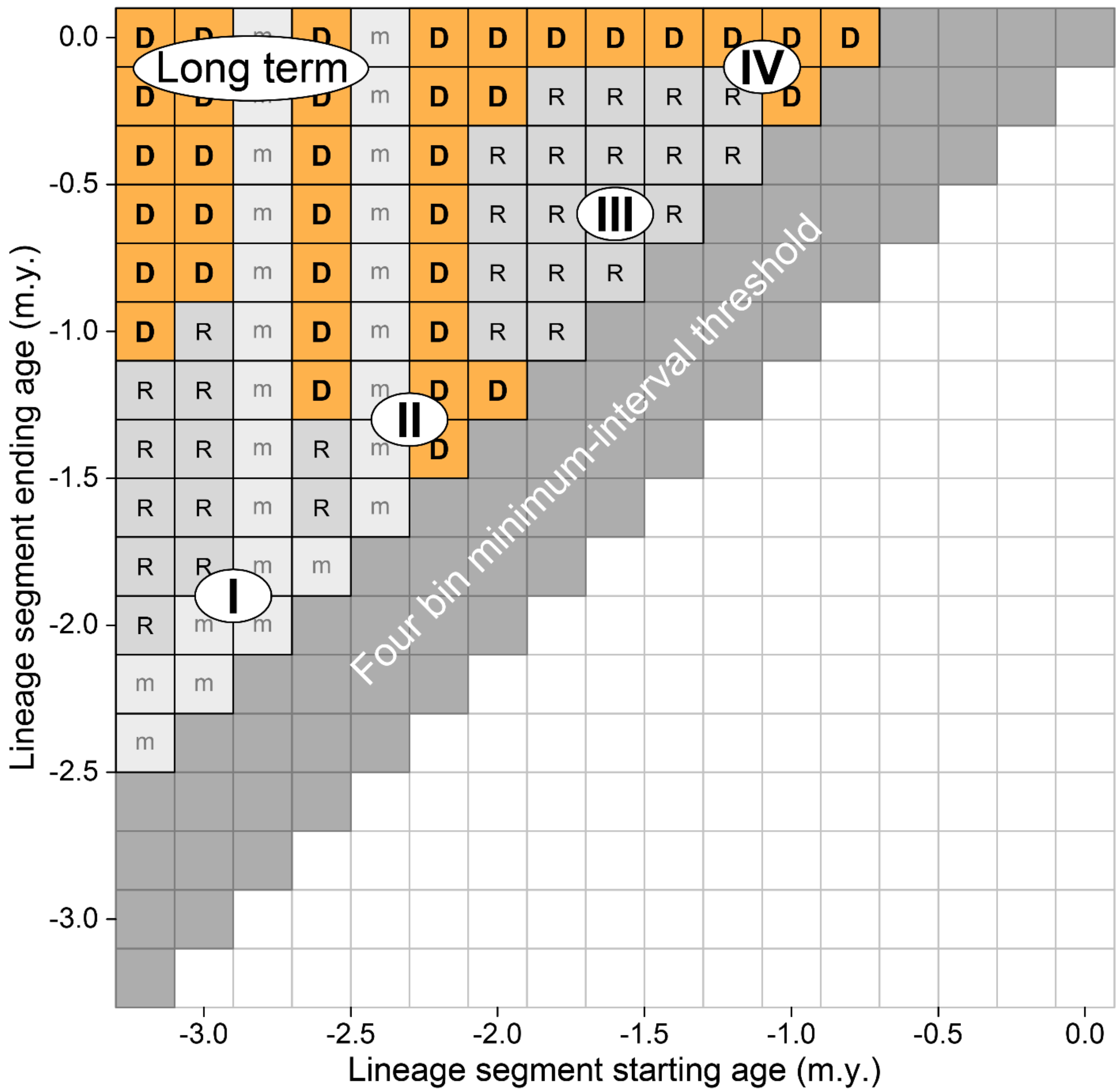

Supplementary Figure S19. Evaluation of evolutionary mode for all time series segments in the binned and smoothed *Australopithecus–Homo* lineage shown above in Supplementary Figure S18. Specimens were binned by age into successive 0.2 m.y. (200 k.y.) bins. Starting age for a segment is shown on the abscissa and ending age is shown on the ordinate. Empty bin segments are marked 'm' for missing. Each segment was analyzed following the protocol illustrated in Figure 1b of the main text. Time series segments with LRI temporal scaling slopes that cannot be distinguished from −0.500 expected for random change are marked 'R'. Time series segments that can be distinguished statistically from stasis and random change but not from directional change are shown in yellow ('D'). When aggregated in 200 k.y. bins no stationary segments can be distinguished from random or directional change. Minimum segment length required for statistical significance is five 200-k.y. bins. Note that time series phases I through IV identified in the main text emerge as distinct modes, but only II and IV can be distinguished from random. Long-term change is an average of change on shorter time scales.
