# Supplementary material for: Pattern and rate in the Plio-Pleistocene evolution of modern human brain size
Source: Sci Rep. 2022 Jul 2;12:11216. doi: 10.1038/s41598-022-15481-3 (PMC9250492; doi:10.1038/s41598-022-15481-3)
Supplement: Supplementary file 4 — Supplementary Table 1. [file 41598_2022_15481_MOESM4_ESM.pdf]

Supplementary Table 1.

Summary statistics describing the overall change of hominin brain size for each of the fourteen time series analyzed here (see Supplementary Information). Consensus calculations are described in the main text, and the consensus pattern of change is illustrated in Figure 1 of the main text.

Study	Figure	Specimens (*N*)	Sample ages (*n*)	Age range (m.y.)		Interval (m.y.)	Regression of ln ECV on age			Long-term rate (std. dev./gen.)	Interval (gen.)
				Max.	Min.		Slope (SE)	Intercept (SE)	r^2		
1. Beals et al. (1984)	S1	99	48	−2.500	−0.010	2.490	0.454 (0.045)	7.254 (0.033)	0.684	0.000113	99,600
2. Aiello and Dunbar (1993)	S2	75	37	−3.000	−0.030	2.970	0.421 (0.027)	7.251 (0.030)	0.871	0.000105	118,800
3. Stanyon et al. (1993)	S3	110	42	−3.300	−0.010	3.290	0.427 (0.022)	7.245 (0.026)	0.906	0.000107	131,600
4. Ruff et al. (1997)	S4	107	38	−1.800	−0.010	1.790	0.386 (0.040)	7.286 (0.027)	0.721	0.000097	71,600
5. D'Amore et al. (2001)	S5	164	61	−3.200	−0.010	3.190	0.415 (0.016)	7.293 (0.016)	0.922	0.000104	127,600
6. DeMiguel and Henneberg (2001)	S6	200	71	−3.200	−0.010	3.190	0.425 (0.018)	7.272 (0.019)	0.888	0.000106	127,600
7. Lee and Wolpoff (2003)	S7	94	17	−1.800	−0.050	1.750	0.356 (0.041)	7.210 (0.041)	0.834	0.000089	70,000
8. Holloway et al. (2004)	S8	131	59	−3.180	−0.015	3.165	0.391 (0.017)	7.252 (0.019)	0.904	0.000098	126,600
9. Ash and Gallup (2007)	S9	109	50	−1.900	−0.035	1.865	0.394 (0.029)	7.248 (0.026)	0.795	0.000099	74,600
10. Bailey and Geary (2009)	S10	170	66	−1.890	−0.010	1.880	0.402 (0.024)	7.274 (0.018)	0.813	0.000101	75,200
11. Shultz et al. (2012)	S11	174	88	−3.200	−0.010	3.190	0.415 (0.016)	7.284 (0.018)	0.890	0.000104	127,600
12. Schoenemann (2013)	S12	166	74	−3.180	−0.010	3.170	0.382 (0.017)	7.248 (0.018)	0.870	0.000095	126,800
13. Du et al. (2018)	S13	75	46	−3.220	−0.345	2.875	0.374 (0.025)	7.192 (0.041)	0.834	0.000094	115,020
14. Ponce de León et al. (2021)	S14	36	18	−2.030	−0.113	1.918	0.339 (0.061)	7.154 (0.078)	0.661	0.000085	76,700
**Consensus**	**1**	**233**	**98**	**−3.200**	**−0.010**	**3.190**	**0.416 (0.016)**	**7.253 (0.019)**	**0.876**	**0.000104**	**127,600**
