# Supplementary material for: Pattern and rate in the Plio-Pleistocene evolution of modern human brain size
Source: Sci Rep. 2022 Jul 2;12:11216. doi: 10.1038/s41598-022-15481-3 (PMC9250492; doi:10.1038/s41598-022-15481-3)
Supplement: Supplementary file 5 — Supplementary Table 2. [file 41598_2022_15481_MOESM5_ESM.pdf]

Supplementary Table 2.

Temporal scaling slopes, intercepts, and step rates h_0 for hominin brain size evolution in each of the 14 time series analyzed in the Supplementary Information..

Study	Figure	Sample ages (n)	Rates	Temporal-scaling slope		Slope interpretation	Temporal-scaling intercept		Step rate h_0
				Median	95% conf. int.		Median	95% conf. int.	
1. Beals et al. (1984)	S1	48	1124	−0.804	−0.866* – −0.709**	Stationary-to-random	−0.543	−0.854 – −0.358	0.286
2. Aiello and Dunbar (1993)	S2	37	666	−0.601	−0.695* – −0.460*	Random	−1.231	−1.820 – −0.874	0.059
3. Stanyon et al. (1993)	S3	42	860	−0.520	−0.616* – −0.472*	Random	−1.579	−1.782 – −1.193	0.026
4. Ruff et al. (1997)	S4	38	702	−0.799	−0.872* – −0.684**	Stationary-to-random	−0.557	−0.967 – −0.329	0.278
5. D'Amore et al. (2001)	S5	61	1829	−0.756	−0.830* – −0.702**	Stationary-to-random	−0.742	−0.935 – −0.509	0.181
6. DeMiguel and Henneberg (2001)	S6	71	2484	−0.797	−0.851* – −0.741**	Stationary-to-random	−0.551	−0.748 – −0.364	0.281
7. Lee and Wolpoff (2003)	S7	17	136	−0.484	−0.642* – −0.386*	Random	−1.868	−2.294 – −1.189	0.014
8. Holloway et al. (2004)	S8	59	1708	−0.806	−0.895* – −0.675**	Stationary-to-random	−0.548	−1.048 – −0.229	0.283
9. Ash and Gallup (2007)	S9	50	1225	−0.835	−0.974* – −0.690**	Stationary-to-random	−0.398	−0.940 – 0.116	0.400
10. Bailey and Geary (2009)	S10	66	2145	−0.772	−0.820* – −0.706**	Stationary-to-random	−0.684	−0.925 – −0.520	0.207
11. Shultz et al. (2012)	S11	88	3825	−0.710	−0.777* – −0.676**	Random-to-stationary	−0.878	−1.005 – −0.644	0.132
12. Schoenemann (2013)	S12	74	2695	−0.744	−0.803* – −0.677**	Random-to-stationary	−0.761	−1.016 – −0.550	0.173
13. Du et al. (2018)	S13	46	1034	−0.798	−0.902* – −0.597**	Stationary-to-random	−0.514	−1.348 – −0.094	0.306
14. Ponce de León et al. (2021)	S14	18	153	−0.899	−1.020 – −0.748**	Stationary	−0.090	−0.717 – +0.356	0.814
**Consensus (N = 233 specimens)**	**1**	**98**	**4747**	**−0.712**	**−0.780* – −0.671***	**Random-to-stationary**	**−0.819**	**−0.981 – −0.569**	**0.152**

*Significantly different from stationary (slope −1.000) but not random change; or significantly different from directional change (slope 0.000) but not random

**Significantly different from random change (slope −0.500) and from directional change (slope 0.000)
